# Quality criteria for pediatric oncology centers: A systematic literature review

**DOI:** 10.1002/cam4.6452

**Published:** 2023-08-16

**Authors:** Sarah P. Schladerer, Maria Otth, Katrin Scheinemann

**Affiliations:** ^1^ Faculty of Health Sciences and Medicine University of Lucerne Lucerne Switzerland; ^2^ Pediatric Hematology‐Oncology Center Children's Hospital of Eastern Switzerland St Gallen Switzerland; ^3^ Department of Oncology, Hematology, Immunology, Stem Cell Transplantation and Somatic Gene Therapy University Children's Hospital Zurich‐Eleonore Foundation Zurich Switzerland; ^4^ Department of Pediatrics McMaster Children's Hospital and McMaster University Hamilton Canada

**Keywords:** cancer management, clinical guidelines, neoplasms, pediatric cancer

## Abstract

**Introduction:**

Survival of children and adolescents diagnosed with cancer improved over the last decades due to better diagnostics, treatment, and supportive care. Quality criteria that measure, compare, and make the quality of care of individual pediatric oncology centers more transparent are heterogeneous and inconsistent.

**Aim:**

With this systematic review, we aimed to summarize existing quality criteria for pediatric oncology centers in countries with highly developed health‐care systems.

**Methods:**

We searched three databases for publications, and websites for guidelines about quality criteria for pediatric oncology centers in February 2022. We considered all types of publications except expert opinions. We excluded publications not focusing on highly developed health‐care systems, addressing the certification of professionals, or focusing on subspecialties (e.g., pediatric neuro‐oncology). We discarded quality criteria if they were too specific (e.g., for a specific treatment center), too broad (e.g., national 5‐year overall survival), or if the aspect was covered by standardized clinical procedures or at the national level. We grouped the identified criteria thematically.

**Results:**

We identified 18 publications and guideline documents with 530 criteria, of which 201 fulfilled the inclusion criteria. The combination of similar criteria resulted in 90 overarching criteria, which we assigned to the following categories: facilities and networks, multidisciplinary team and other experts, supportive care, treatment, long‐term care, and volume and numbers.

**Conclusion:**

Our results provide a comprehensive overview of existing quality criteria for pediatric oncology in countries with highly developed health‐care systems. These criteria can serve as a basis to develop national quality criteria in pediatric oncology.

## 1 INTRODUCTION

The survival of children and adolescents diagnosed with cancer increased markedly in countries with highly developed health‐care systems.^1^, ^2^, ^3^ The 5‐year survival rates over all diagnostic categories reached ≥85%, for example, in the United States,^4^ in Germany,^5^ and in Switzerland.^2^ These survival rates reflect achievements and improvements in diagnostics, treatment, and supportive care but do not provide direct information about the quality of care delivered (e.g., rate of central venous line infections) by single treatment centers. This is, however, an important factor for the treatment centers themselves but also for the health‐care systems, insurances, and most importantly, for the patients. Objectifiable and well‐measurable quality criteria are necessary for this purpose.

Such quality criteria make treatment centers comparable (within and between countries), enable repeated assessments of individual centers over time, and assessable (e.g., monitoring based on the defined quality criteria) for the quality of care they provide. The extent of fulfillment of quality criteria by treatment centers can be an orientation for health‐care professionals, health sector personnel less familiar with pediatric oncology (e.g., insurances), and laypersons (e.g., parents, patients, survivors). In addition, politicians and policymakers may rely on such information when establishing new laws, national care standards, or public health programs related to pediatric oncology.

Quality criteria are defined as aspects or components of processes, outcomes, or care structures that affect the quality of care.^6^ Quality criteria should be measurable, and their definition should be as clear that one can determine whether they are present or absent.^7^, ^8^ Using quality criteria increases transparency, reflects the current standard of care nationally and internationally, and favors further improvements in the quality of care delivered.

Quality criteria for pediatric oncology centers should consider the specific aspects of pediatric cancer and pediatric patients. Besides diagnoses and treatment approaches, also the social aspects differ immensely between children and adults with cancer. Families and parents have a more active role when a child is diagnosed with cancer. In addition, the child's normal development and education are important aspects to be considered during and after treatment.

Different approaches already exist to measure quality in pediatric oncology. The German Cancer Society (Deutsche Krebsgesellschaft, DKG) offers a tool to certify German‐speaking pediatric oncology centers.^9^, ^10^ The European Society for Paediatric Oncology (SIOPE) provides a guideline document for European pediatric oncology centers on standards of care for children with cancer.^11^ Additional guidelines exist for the United States and the United Kingdom.^12^, ^13^, ^14^ In addition, different research groups developed and suggested specific quality criteria.

In this systematic review, we summarize the current evidence on quality criteria for pediatric oncology centers in countries with highly developed health‐care systems. We considered countries with highly developed health‐care systems to be those with good overall scores for life expectancy, avoidable mortality, population coverage, financial protection, service coverage, effective primary, preventive, and secondary care, health spending, and the number of physicians, nurses, and hospital beds as indicated by the core indicators of the “Health at Glance 2021” report.^15^

## 2 METHODS

### 2.1 Search strategy

On February 21 and 22, 2022, we systematically searched the databases PsycINFO, PubMed, and CINAHL for all types of publications published since 2000, and written in English or German. The search strategy included three concepts: quality and certification criteria, children and adolescents, and oncology (Appendix 1). We created a PubMed alert to identify newly published publications until mid of May 2022, tracked references of included publications, and checked related websites (Appendix 2). We followed the PRISMA 2020 guideline for reporting systematic reviews^16^ and preregistered this systematic review on PROSPERO (https://www.crd.york.ac.uk/prospero/display_record.php?ID=CRD42022308185).

### 2.2 Selection of eligible publications and quality criteria

After merging the database search results, we identified and eliminated duplicate records manually (software Endnote; webtool Rayyan [https://rayyan.ai/, RRID:SCR_017584]).^17^ Two researchers (SSch and MO) independently screened titles and abstracts, full texts, and quality criteria. In case of disagreement, we consulted a third researcher (KS). We used Rayyan (https://rayyan.ai/, RRID:SCR_017584)^17^ for the title and abstract screening. We included all types of publications that mentioned quality criteria in pediatric oncology except for expert opinions. At this stage, we excluded publications focusing on adults, on the certification of professionals (e.g., nurses), or publications not targeting highly developed health‐care systems. At the full‐text stage, we excluded publications with <75% of patients aged ≤18 years, <75% of patients diagnosed with cancer, publications that did not clearly define, mention or apply quality criteria, publications addressing laboratory procedures or subspecialties of pediatric oncology (e.g., radiotherapy), and publications that referred to criteria not measuring the quality of treatment centers (e.g., national 5‐year survival).

Prior to data extraction, we screened the quality criteria listed in eligible publications. In accordance with exclusion criteria for publications, we did not consider quality criteria explicitly referring to adolescents and young adults (per definition >75% aged >18 years). We discarded quality criteria if they were too specific (e.g., for a specific treatment center or diagnosis), too broad (e.g., not clear how to measure the fulfillment of criteria objectively), or if they did not refer to the quality of a single center (e.g., national 5‐year survival). We excluded criteria if they were covered by good clinical practice, clinical trial participation or treatment protocols (e.g., demonstration of adherence to the European Union Directive on Good Clinical Practice). We further excluded criteria if the addressed aspects were regulated nationally or if the criteria were specific for the certification of professionals or subspecialties (e.g., rehabilitation, palliative care, and bereavement). However, we considered the existence of subspecialties or access to them as criteria.

### 2.3 Data extraction and critical appraisal

We extracted quality criteria and publication characteristics using standardized data extraction forms. One researcher (SSch) performed the data extractions and quality assessments of included publications, and a second researcher (MO) verified them. If criteria were reported for a specific context, we generalized them (e.g., “Included cases in treatment optimization studies of the German Society for Pediatric Oncology and Hematology (GPOH)”^9^ to “Number/Proportion of clinical trial participation”). We summarized the quality criteria thematically. Since we did not aim to summarize how the criteria can be measured, we did not extract this information.

We used the critical appraisal tools from the Joanna Briggs Institute (JBI)^18^ to assess the selected publications' quality and risk of bias. We applied the critical appraisal checklists for cross‐sectional, cohort, and qualitative studies, and for systematic reviews. Each checklist consists of eight to eleven criteria depending on the type of publication. If a publication used different methodological approaches (e.g., systematic review and qualitative part), we applied all respective checklists. As the JBI does not provide a rating scale for publication quality, we defined three quality categories. Criteria judged as “not applicable” were not considered in the quality assessment. We defined “Quality 1” if publications met all criteria, “Quality 2” if publications did not meet one or two criteria of the respective checklist, and “Quality 3” if publications did not meet three or more criteria (Table 1, Data S1).

**TABLE 1:** Included publications and guideline documents (*n* = 18) reporting quality criteria for pediatric oncology.

First author/publisher, year, place	Publication design (method)	Outcome	Context or target group	Quality assessment^a^ ^,^ ^b^
Publications				
Institute for Quality and Efficiency in Health Care (IQWiG), 2005, Germany^44^ and IQWiG, 2009, Germany^45^	Report (systematic literature and clinical practice guidelines search)	Patient‐relevant outcomes: survival, treatment‐related death, health‐related quality of life, pain, and long‐term consequences of the disease and therapy Outcomes for procedures and infrastructure: information on standards and clinical practice guidelines, particular features of quality indicators, and organizational requirements for psychosocial support and rehabilitation	Quality of care assessment for pediatric oncology patients (acute leukemia, malignant lymphomas, and brain tumors)	SR: Quality 2
Bradley, 2013b, Canada^24^	Systematic review (review of quality assessment frameworks and gray literature search; focus group to provide provisional quality indicators)	33 Quality indicators for pediatric oncology systems	Pediatric oncology system	SR: Quality 3
Bradley, 2013a, Canada^25^	Qualitative study (mailed survey, modified Delphi Panel Consensus Meeting)	20 Quality indicators for pediatric oncology systems	Pediatric oncology system	QR: Quality 1
Corey & Snyder, 2008, US^19^	Report (plan‐do‐study‐act cycle)	Time to antibiotics (TTA) (door/fever‐to‐antibiotic delivery time)	Pediatric cancer patients with febrile neutropenia	NA
de Rojas, 2019, Spain^39^	Retrospective cohort study (retrospective database record analysis, review of existing QIs in other areas of oncology, national, and consensus documents)	34 Quality indicators for pediatric CNS tumors How defined quality indicators are met in the audit; outcome given by the quality indicators for pediatric CNS tumors	Pediatric medulloblastoma patients	CS: Quality 3
Fletcher, 2013, US^22^	Retrospective cohort study (retrospective patient record analysis, TTA measured as a continuous variable and in 60 min intervals)	Association of TTA administration with outcomes of febrile neutropenia in pediatric oncology patients. Outcomes: inhospital mortality, pediatric intensive care unit admission within 24 h of presentation, fluid resuscitation 40 mL/kg within 24 h of presentation, length of stay	Pediatric oncology patients with febrile neutropenia	CS: Quality 2
Knops, 2012, The Netherlands^29^	Literature review and qualitative study (RAND modified Delphi method)	Recommendations for “process” and “structure” of medical care in pediatric oncology	Pediatric oncology patients	SR: Quality 3 QR: Quality 2
Knops, 2013, The Netherlands^23^	Systematic review (database search and reference tracking)	Quality of care or survival in childhood cancer	Hospital volume, pediatric oncology patients	SR: Quality 1
McCavit & Winick, 2012, Canada and US (survey within the Children's Oncology Group)^21^	Brief report (electronic survey)	Proportion of centers tracking TTA as a quality‐of‐care measure, respondents' TTA benchmark/standard, locations where TTA is collected (inpatient unit, outpatient clinic, or emergency department), most recent TTA data from each respondent	Pediatric oncology patients with febrile neutropenia	CSS: Quality 2
Olshefski, 2020, US^20^	Original article (development and test of Cancer Care Index; retrospective and real‐time, multidisciplinary microsystem‐based teams addressed specific aims for each domain)	Cancer Care Index and the impact of its application on quality and safety performance	Oncology‐bone marrow transplant service	NA
Teichman, 2017, Canada^46^	Literature search, qualitative study (de novo development by a steering committee, Delphi process)	Quality metrics for a leukemia–lymphoma clinic	Pediatric patients diagnosed with leukemia or lymphoma, outpatient setting	QR: Quality 2
ten Berg, 2018, The Netherlands^47^	Qualitative study, retrospective cohort study (consensus process questionnaire, patient records)	Seven structure, process, and outcome indicators developed based on Dutch Childhood Oncology Group guidelines	Pediatric oncology patients with febrile neutropenia	QR: Quality 3 CS: Quality 3
Guideline documents				
National Institute for Health and Care Excellence (NICE), 2005, UK^13^	Guidance on cancer service—the Manual		Pediatric oncology	NA
NICE, 2014, UK^14^	Quality standard		Pediatric oncology	NA
Federal Joint Committee (G‐BA), 2021, Germany^48^	Guideline		Pediatric oncology	NA
German Cancer Society (DKG), 2021, Germany^9^	Certification survey form		Pediatric oncology	NA
The European Society for peadiatric oncology (SIOPE); Kowalczyk, 2009, Europe^11^	Standards of care		Pediatric oncology	NA
American Academy of Pediatrics (AAP); Hord, 2014, US^12^	Policy statement: Guidelines for Pediatric Cancer Centers		Pediatric oncology	NA

Abbreviations: AAP, American Academy of Pediatrics; DKG, German Cancer Society (Deutsche Krebsgesellschaft); G‐BA, Federal Joint Committee (Gemeinsamer Bundesausschuss); IQWiG, Institute for Quality and Efficiency in Health Care (Institut für Qualität und Wirtschaftlichkeit im Gesundheitswesen); NICE, National Institute for Health and Care Excellence; SIOPE, The European Society for peadiatric oncology; TTA, time to antibiotics.

^a^ Abbreviations for the type of critical appraisal tools used for quality assessment: SR checklist for systematic reviews, QR checklist for qualitative research, CS checklist for cohort studies, CSS checklist for analytical cross‐sectional Studies, NA not applicable.

^b^ For details, see Data S1.

## 3 RESULTS

**FIGURE 1:**
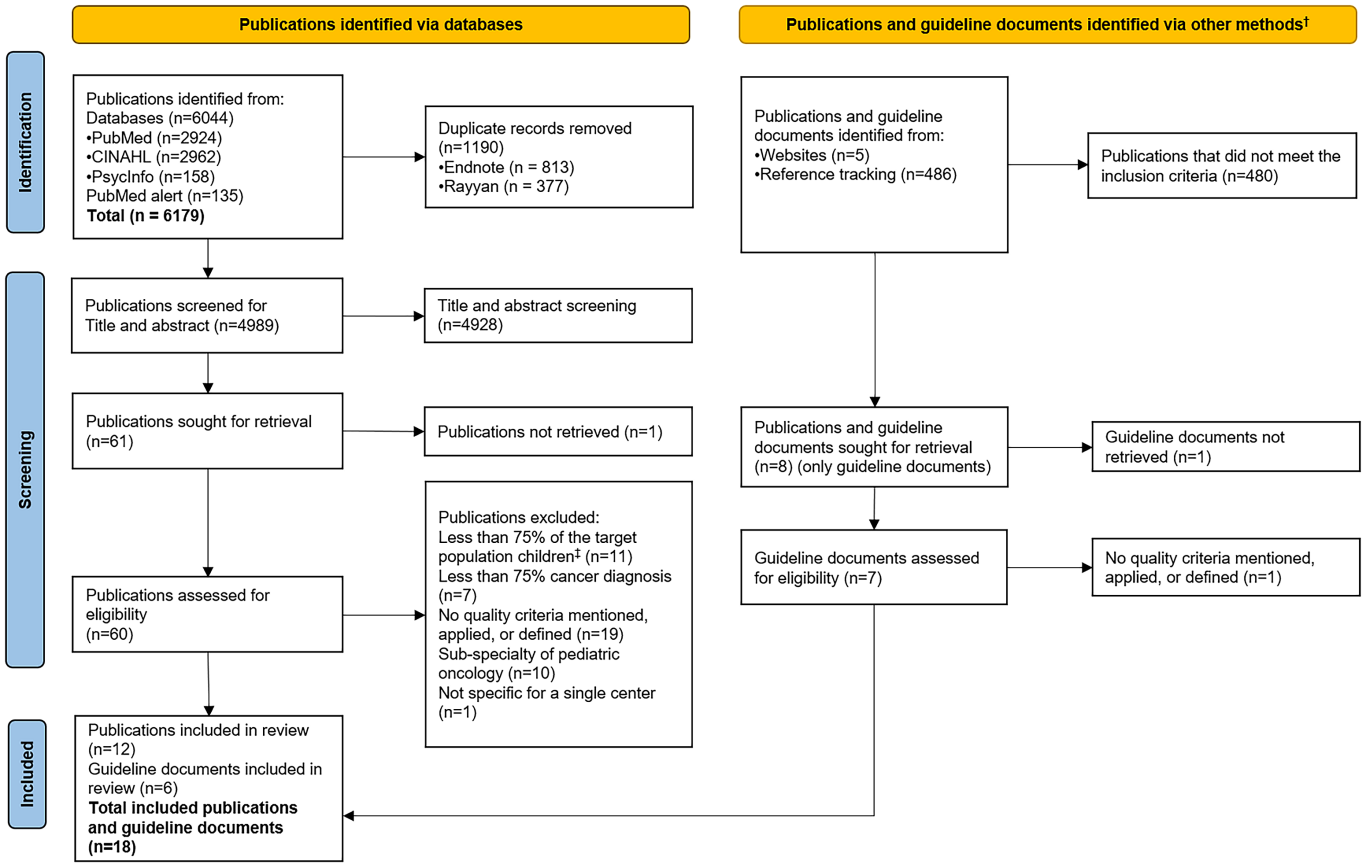
PRISMA 2020 flow diagram of included publications and guidelines.^16^ ^†^Reference tracking of included publications and searching related websites. ^‡^Children defined as ≤18 years of age.

We identified 6179 publications from the three databases, out of which 1190 duplicates were identified and removed. The title and abstract screening resulted in 61 publications, of which 12 remained after the full‐text screening. The gray literature search considered 486 additional publications, guideline documents, and websites, six of which we included in the review, resulting in a total of 18 publications/guideline documents (Figure 1, Table 1).

The most frequent reasons for exclusions at the full‐text stage were: (1) publications did not mention, apply or define quality criteria, (2) <75% of the target group was aged ≤18 years, and (3) publications addressed subspecialties only (Figure 1). Two publications were assigned to “Quality 1”, three to “Quality 2”, and four to “Quality 3”. One publication used different methodological approaches (qualitative and cohort study), where one tool indicated “Quality 2” and one “Quality 3”. We did not assess the quality of the six guideline documents and two publications^19^, ^20^ as their design did not fit any of the available checklists from the JBI critical appraisal tools (Table 1). The main reason for a reduced quality of systematic reviews was that it was unclear whether critical appraisal of included publications was conducted independently by two or more reviewers. The main reason for cohort studies was the uncertainty whether exposure was measured validly and reliably, and uncertainty whether there was congruity between the research methodology and the methods used to collect data for qualitative publications (Data S1).

The included publications comprised 530 quality criteria, of which we excluded 329 (62%). The main reasons were: (1) criteria were too broad (e.g., not clear how to measure the fulfillment of criteria objectively; 34%), (2) criteria were too specific (e.g., for a specific treatment center; 23%), or (3) that criteria did not measure the quality of care in general or for individual centers (18%) (Appendix 3).

Finally, 201 quality criteria fulfilled the inclusion criteria (Data S2). The detail accuracy differed between criteria from the different publications. The thematical grouping carried out for this reason resulted in a final set of 90 overarching criteria belonging to the following six categories: facilities and networks (*n* = 18), multidisciplinary team (MDT) and other experts (*n* = 35), supportive care (*n* = 20), treatment (*n* = 12), long‐term care (*n* = 4), and volume and numbers (*n* = 1) (Table 2). Publications suggested relevant threshold values for some quality criteria. For the criterion “time to antibiotic (TTA) administration” in patients with febrile neutropenia (“Supportive Care” category), publications mentioned benchmarks of 30^19^, ^21^ or 60 min.^20^, ^21^, ^22^ For the criterion “number of cases per year and provider/clinic”, publications suggested ≥5 cases/year/provider as a high volume^23^ or 30 new cases/year/unit as a minimum.^11^ Stated thresholds for other criteria are listed in the table of included quality criteria in their original detailed version (Data S2).

**TABLE 2:** List of summarized quality criteria for pediatric oncology centers by thematical categories.

Facilities and networks		
Access to the following important facilities		
Pharmacy^12^, ^29^, ^48^	Microbiology Institute^48^	Pediatric cardiology^48^
Adult hematology and oncology^48^	Laboratories^11^, ^12^, ^29^, ^48^: hematology, hematopathology, clinical chemistry, transfusion	Pathology^29^, ^48^
Orthopedics^48^	Pediatric intensive care unit^29^, ^48^	Pediatric radiology^12^, ^29^, ^48^
Stem cell transplant unit^12^	Pediatric nephrology^12^, ^29^, ^48^	Radiation therapy^12^, ^20^, ^29^, ^48^
Pediatric surgery^29^, ^48^	Pediatric neurosurgery^13^, ^29^, ^48^	Nuclear medicine^48^
Pediatric anesthetics^29^	Hospital hygiene^48^	Childhood cancer registry^9^, ^11^, ^29^

Multidisciplinary team (MDT) and other experts		
MDT established, including regularly scheduled MDT conferences^9^, ^11^, ^12^, ^13^, ^29^, ^48^	Number of pediatric oncology disciplines with multidisciplinary staffing ratios for pediatric oncology^13^, ^24^, ^25^, ^29^, ^48^	
An MDT should consist of representatives from the following disciplines/expertise (disciplines involved depend on the patients' needs)		
Pediatric oncology practitioner‐in‐charge/lead clinician (also with expertise in late effects)^13^, ^29^	Pediatric oncologists^11^, ^12^, ^13^, ^48^	Pediatric oncology nurses^11^, ^12^, ^13^, ^48^
Pediatric radiologists^12^, ^13^, ^29^	Pediatric surgeons^12^, ^13^, ^29^	Radiation oncologists^12^, ^29^
Pediatric endocrinologist^12^, ^13^	Pediatric critical care specialists^12^, ^29^	Pediatric infectious diseases specialists^12^
Pediatric anesthesiology^12^, ^29^	Pediatric cardiologist^12^, ^29^	Pediatric neurologist^12^, ^29^
Pediatric gastroenterologist^12^, ^29^	Pediatric nephrologist^12^, ^29^	Pediatric pulmonologist^12^, ^29^
Ear–nose–throat specialist^29^	Ophthalmologist^29^	Long‐term care (experts)^29^
Genetics specialists^12^, ^29^	Pain management experts^29^	Palliative care specialists^12^, ^13^, ^29^
Complementary and alternative therapies^12^	Dieticians^11^, ^12^, ^48^	Occupational therapists^11^, ^48^
Rehabilitation specialists^11^, ^12^	Pharmacists experienced in chemotherapy preparation^12^, ^13^	Laboratory technicians^11^
Medical secretaries and data managers^11^	Dentist^12^	Psychosocial care/services^11^, ^12^, ^13^, ^29^, ^48^
Ward teachers^11^, ^12^, ^13^	Activity/play therapy staff^11^, ^13^, ^29^	Pediatric pathologist^12^, ^13^, ^29^

Supportive care		
Central venous catheter (CVC)		
Complication rates: particularly the incidence of CVC‐associated infection^13^, ^24^	Surgical complication rates (failure to insert the desired device or leaving the catheter tip in an unacceptable location) related to CVC placement^20^	Written policies/ procedures for the management of CVC^24^, ^25^
Existence of supportive care guidelines^24^, ^25^, ^29^ including supportive care (guidelines) for		
Nausea, vomiting and bowel disturbance^13^, ^24^, ^25^	Nutritional assessment^13^, ^20^, ^24^, ^29^	Fertility (preservation) discussion^14^, ^20^, ^29^
Palliative care (including bereavement)^11^, ^13^, ^24^, ^29^, ^39^	(Neuro‐) Rehabilitation^11^, ^13^, ^14^	Dental care^13^
Pain relief, including local protocol for pain relief procedures and adequate pain management^13^, ^24^, ^25^, ^44^, ^45^, ^46^	Psychological or psychosocial care, including provision of/information about social care^9^, ^11^, ^13^, ^14^, ^20^, ^44^, ^45^, ^48^	Provision of school education^11^, ^20^, ^29^
Provision of cancer education^12^		
Febrile neutropenia (F&N)		
Guidelines on how to approach a child with F&N (availability, risk‐stratified approach, escalation for fever persistence)^13^, ^24^, ^25^, ^47^	Number/proportion of clinical F&N episodes in which the patients with or without microbial focus are treated with first line antibiotics according to local guidelines^47^	Number/proportion of clinical F&N episodes in which patients are admitted to ICU^47^
Number/proportion of clinical F&N episodes in which the patient died^47^	Number/proportion of fungal health care‐associated infections^20^	Time to antibiotics (TTA) administration^19^, ^20^, ^21^, ^22^

Treatment		
Number/proportion of patients presented in the interdisciplinary tumor conference (for solid and liquid tumors separately or combined)^9^, ^12^, ^24^, ^48^	Protocol compliance (e.g., number of major clinical trial protocol violations)^13^, ^24^, ^39^	Number/proportion of clinical trial participation^9^, ^13^, ^14^, ^24^, ^25^, ^29^, ^39^
Number/proportion of refusal and failure to complete treatment^13^		
Delay in/ wait time to start of		
Radiotherapy^13^	Chemotherapy^13^, ^24^, ^25^	First therapeutic intervention^24^, ^25^
Release of pathology results^13^, ^24^, ^25^		
Medication		
Number/proportion of patient safety incidents related to chemotherapy prescriptions^14^	Number/proportion of actual drug or dose errors identified for patients on active treatment^13^, ^24^, ^25^	Number/proportion of potential drug or dose errors identified for patients on active treatment^13^, ^24^, ^25^
Number/Proportion of elective pediatric oncology ambulatory procedures requiring anesthesia that are deferred to the next day or beyond due to resource limitation(s)^24^, ^25^		

Long‐term care		
Number/proportion of survivors of childhood cancer with a survivor care plan^13^, ^14^, ^24^, ^25^, ^29^	Number/proportion of survivors who have their survivorship care plan reviewed 5 years after the end of treatment^14^	Established follow‐up structure^11^, ^13^, ^24^, ^25^
Established transition structure^12^, ^29^		

Volume and numbers		
Number of cases per year and provider/clinic^9^, ^11^, ^23^, ^39^		

## 4 DISCUSSION

We could identify 90 quality criteria belonging to six thematical categories. Even though the identified criteria were heterogeneous, for example, related to the scope (some criteria for specific centers or disciplines only), they can serve as a basis for developing uniform and harmonized quality criteria for pediatric oncology centers in different countries.

Bradley et al. identified a set of quality criteria specific to Canadian pediatric oncology.^24^, ^25^ Many of their criteria refer to the Pediatric Oncology Group of Ontario (POGO) and to their satellite system institutions. POGO is a nonprofit organization (and the official advisor to the Ontario Ministry of Health on childhood cancer care and treatment) and works to ensure that everyone with pediatric cancer has access to the best care and support.^26^ Even though the quality criteria of this publication focus on the POGO system, we included its results in a generalized form.

Though heterogeneous, the criteria of the different publications in all six categories have also resembled and complemented each other. For the criteria on facilities and networks, eligible publications provided different levels of detail of single facilities but finally did not differ much regarding the main components. Even though we focused on general quality criteria, some might not be relevant for pediatric oncology centers in certain countries. For example, the criterion on reporting to the “childhood cancer registry” is only applicable for centers where cancer registration is not common practice. In countries where pediatric cancer registries cover the national population (e.g., Hungary, Greece, Germany, France, Belarus, Czech Republic, and the United Kingdom^27^) or registration is mandatory (e.g., Switzerland^28^), the fulfillment of this criterion might be less meaningful.

Most criteria related to MDT addressed disciplines and specialists with expertise in the pediatric field that should be part of these teams. As children and adolescents have different needs than adults, one should consider the pediatric competence of MDT members when defining quality criteria. Even though not stated explicitly in the included publications, the expertise of an MDT differs depending on the patients' needs and underlying diagnosis. Further, an MDT should be led by a person responsible for and coordinating the different involved disciplines.^13^, ^29^ The content of protocols from MDT meetings was not specified, which might be a relevant quality criterion too.

Quality criteria on supportive care covered the topics of specific supportive care disciplines, guidelines (e.g., for rehabilitation), central venous catheters (CVC), and febrile neutropenia. Two publications mentioned the “CVC‐associated infection rate” as a quality criterion.^13^, ^24^ This is an important measure in daily clinical practice, and additional publications examined different improvement approaches to prevent and reduce CVC‐associated infections.^30^, ^31^, ^32^ Duffy et al. examined, for example, the effect of a CVC care bundle consisting of several tasks (e.g., standardized hand hygiene or change of dressings) on the frequency of CVC‐associated infections.^31^ “TTA administration” seems to be another well‐established criterion for pediatric oncology patients with febrile neutropenia. Besides the included publications mentioning TTA as a quality criterion, several researchers addressed how to reduce TTA in patients with febrile neutropenia in inpatient,^33^ intensive care,^34^ or emergency departments.^35^, ^36^, ^37^ When using TTA as a quality criterion, it is essential to define a clear starting point. Taking the time from first measured fever might be difficult in the outpatient setting as the time to the hospital differs between patients, depending on the living distance from the hospital. The resulting difference in travel time to the hospital has an impact on the TTA, which cannot be influenced by the quality of the center itself. The starting point on admission to the hospital (emergency room or ward) would therefore be more indicative of the quality of the pediatric oncology center.

Quantifying “Fungal Health Care‐Associated Infections” in patients with febrile neutropenia was another quality criterion.^20^ This is a relevant measure as invasive fungal infections are dangerous for immunocompromised patients.^38^ However, a general monitoring system for all pathogens, including viral, bacterial, and fungal infections, could be favored over the monitoring of fungal pathogens only. Knowing the local microbiological spectrum can help pediatric oncology centers in the selection of the appropriate empiric antibiotic treatment in case of febrile neutropenia. It may further help to identify local environmental factors (e.g., an increase in invasive aspergillosis in areas with construction work). Both factors increase the quality of care.

In the category “treatment”, one could favor time‐related criteria. However, defining generalized thresholds, for example, for the criteria “first therapeutic intervention” or the “release of the pathology results” could be problematic as the time taken depends strongly on the diagnosis and required analyses. While the results for leukemia can be provided relatively quickly, it requires more time for bone tumors, where the tissue must first be decalcified. Such aspects need to be considered when assessing the treatment quality of a center with time‐related criteria and might result in many separate criteria.

Different publications mentioned the “number of cases per year and provider/clinic”^9^, ^11^, ^23^, ^39^ in the category volume and numbers. However, it is unclear whether a higher volume indicates better quality of care. A retrospective cohort study found no association between a low case volume and increased mortality or intensive care unit (ICU) admission in pediatric acute lymphoblastic leukemia patients.^40^ Another publication also did not find a difference in survival between centers of bigger and smaller sizes for pediatric neuro‐oncology patients.^41^ Besides, by measuring the quality of care quantitatively, relevant qualitative aspects, such as the provision of various supportive care services that contribute to the quality of care, are neglected. Previous research also questioned the evidence of thresholds and the generalizability of using patient numbers for assessing the quality of care in Germany.^42^

Many pediatric oncology centers are grown historically, for example, based on geographic location or because pediatric oncologists initially worked there, but not based on the provided quality. Therefore, quality criteria can be used as an orientation in monitoring the existing centers, but also to increase or decrease the number of centers, depending on the current national situation. For example, centers can check which facilities should be established, which representatives an MDT should consist of, and which supportive care areas they should cover. Audits can check the fulfillment of quality criteria when assessing pediatric oncology centers. For accreditation of centers, the quality criteria identified in this review could be included in surveillance software or hospital systems for pediatric oncology centers, which ministries of health and other relevant players can access. The extent of compliance to the criteria could be made publicly available and could help different stakeholders to advocate for equal access and care within a country and to address shortcomings on national and political levels. Further, if centers cannot meet quality criteria, health ministries could help to allocate resources to these centers to improve care quality. Overall, applying uniform quality criteria can increase the transparency and comparability of centers within and between countries. However, the list of quality criteria provided in this review needs to be cautiously applied. Differences in health‐care systems between countries necessitate adapting the quality criteria to the national needs and circumstances.

### 4.1 Strengths and limitations

The strength of this review is that we searched three databases and considered almost all publication types published over more than 20 years. In addition, we tracked references and searched gray literature. Further, the title and abstract screening, full‐text screening, data extraction, and quality assessment were performed by two researchers. A limitation inherent to systematic literature reviews is that we might have missed criteria that are considered quality criteria which, however, were not named as such in the literature. The same is true for criteria used at national levels, which are not publicly available. The external validity of this list of quality criteria may be limited by the fact that it applies to countries with highly developed health‐care systems, as quality criteria for countries with less developed health‐care systems need to address more fundamental aspects of care. However, other publications explicitly focus on quality criteria for pediatric oncology in countries with less developed health‐care systems.^43^

## 5 CONCLUSION

The list of quality criteria provided by this systematic review can serve as a basis to develop national sets of quality criteria or assessment tools for pediatric oncology centers in highly developed health‐care systems. To select a relevant subset of criteria adapted to national circumstances, experts should qualitatively discuss and evaluate the criteria and state uniform measurements in future research.

## AUTHOR CONTRIBUTIONS

**Sarah P. Schladerer:** Formal analysis (lead); investigation (lead); methodology (lead); project administration (supporting); visualization (lead); writing – original draft (lead); writing – review and editing (lead). **Maria Otth:** Conceptualization (supporting); formal analysis (supporting); funding acquisition (supporting); investigation (supporting); methodology (supporting); project administration (supporting); supervision (supporting); writing – original draft (supporting); writing – review and editing (supporting). **Katrin Scheinemann:** Conceptualization (lead); formal analysis (supporting); funding acquisition (lead); investigation (supporting); methodology (supporting); project administration (lead); supervision (lead); writing – original draft (supporting); writing – review and editing (supporting).

## FUNDING INFORMATION

Swiss Cancer Research, Grant number: HSR‐5219‐11‐2020.

## CONFLICT OF INTEREST STATEMENT

There are no conflicts of interest for any of the authors.

## Supporting information


Data S1

Data S2:

## Data Availability

Data sharing is not applicable to this article as no new data were created or analyzed in this study.

## APPENDIX 1

### 1.1 Database (PubMed, CINAHL, PsycINFO) search strategy.

Concept 1: Quality and certification criteria.

Concept 2: Children and adolescents.

Concept 3: Oncology.

**Table:**

PubMed Search (performed on February 22nd, 2022)	
#1	“Quality Indicators, Health Care”[MeSH]
#2	quality of care [tiab] OR quality indicator* [tiab] OR quality measure* [tiab] OR quality marker* [tiab] OR quality improvement* [tiab] OR quality appraisal* [tiab] OR certification* [tiab] OR performance indicator*[tiab] OR performance metric* [tiab] OR performance measure* [tiab] OR standard of care [tiab] OR standards of care [tiab]
#3	“Child”[MesH] OR “Pediatrics”[Mesh] OR “Infant”[MesH] OR “Adolescent”[MesH]
#4	child*[tiab] OR infan*[tiab] OR adolescen*[tiab] OR newborn*[tiab] OR baby*[tiab] OR babies[tiab] OR neonat*[tiab] OR perinat*[tiab] OR postnat*[tiab] OR schoolchild*[tiab] OR school child*[tiab] OR kid[tiab] OR kids[tiab] OR toddler*[tiab] OR teen*[tiab] OR boy[tiab] OR boys [tiab] OR girl*[tiab] OR juvenil*[tiab] OR youth*[tiab] OR kindergar*[tiab] OR pediatric*[tiab] OR paediatric*[tiab] OR school*[tiab] OR preschool*[tiab] OR pre school*[tiab] OR elementary school*[tiab] OR highschool*[tiab] OR high school*[tiab] OR schoolage*[tiab] OR school age*[tiab]
#5	“Neoplasms”[MesH] OR “Oncology Service, Hospital”[Mesh] OR “Cancer Care Facilities”[Mesh]
#6	cancer* [tiab] OR oncolog* [tiab] OR neoplasm*[tiab] OR carcinom* [tiab] OR tumor* [tiab] OR tumour*[tiab] OR malignan*[tiab] OR hematooncological [tiab] OR hemato oncologic*[tiab] OR hematologic neoplasm* [tiab] OR hematolo*[tiab]
#7	all [sb] “Animals”[Mesh] NOT “Humans”[Mesh]
#8	“english”[Language]
#9	“german”[Language]
#10	(“2000/01/01”[Date ‐ Publication]: “2022”[Date ‐ Publication])
#11	#1 OR #2
#12	#3 OR #4
#13	#5 OR #6
#14	#11 AND #12 AND #13
#15	#14 NOT #7
#16	#8 OR #9
#17	#15 AND #16
#18	#17 AND #10

### 1.2

**Table:**

CINAHL Search (performed on February 22nd, 2022)	
Search ID#	Search terms
S1	(MH “Quality Assurance+”)
S2	TI ((quality W3 care) OR (quality W1 indicator*) OR (quality W1 measure*) OR (quality marker*) OR (quality improvement*) OR (quality W1 appraisal*) OR (certification*) OR (performance W1 indicator*) OR (performance W1 metric*) OR (performance W1 measure*) OR (standard N3 care) OR (standards N3 care)) OR AB ((quality W3 care) OR (quality N4 indicator*) OR (quality W1 measure*) OR (quality marker*) OR (quality improvement*) OR (quality W1 appraisal*) OR (certification*) OR (performance W1 indicator*) OR (performance W1 metric*) OR (performance W1 measure*) OR (standard N3 care) OR (standards N3 care))
S3	(MH “Child+”) OR (MH “Infant+”) OR (MH “Adolescence+”) OR (MH “Pediatrics+”)
S4	TI ((child*) OR (infan*) OR (adolescen*) OR (new W1 born*) OR (baby) OR (babies) OR (neonat*) OR (perinat*) OR (postnat*) OR (school W1 child*) OR (toddler*) OR (teen*) OR (boy*) OR (girl*) OR (youth*) OR (kindergar*) OR (p#ediatric*) OR (school*) OR (preschool*) OR (pre W1 school*) OR (elementary W1 school*) OR (highschool*) OR (high W1 school*) OR (schoolage*) OR (school W1 age*)) OR AB ((child*) OR (infan*) OR (adolescen*) OR (new W1 born*) OR (baby) OR (babies) OR (neonat*) OR (perinat*) OR (postnat*) OR (school W1 child*) OR (toddler*) OR (teen*) OR (boy*) OR (girl*) OR (youth*) OR (young W2 adult*) OR (kindergar*) OR (p#ediatric*) OR (school*) OR (preschool*) OR (pre W1 school*) OR (elementary W1 school*) OR (highschool*) OR (high W1 school*) OR (schoolage*) OR (school W1 age*))
S5	(MH “Neoplasms+”) OR (MM “Cancer Care Facilities”) OR (MM “Oncology Care Units”) OR (MM “Childhood Neoplasms”)
S6	TI ((cancer*) OR (oncolog*) OR (neoplasm*) OR (carcinom*) OR (tumo#r*) OR (malignan*) OR (hematooncological) OR (hemato W1 oncological) OR (hematologic W1 neoplasm*) OR (hematolo*)) OR AB ((cancer*) OR (oncolog*) OR (neoplasm*) OR (carcinom*) OR (tumo#r*) OR (malignan*) OR (hematooncological) OR (hemato W1 oncological) OR (hematologic W1 neoplasm*) OR (hematolo*))
S7	(MH “Animals+” not MH “Humans+”)
S8	LA German
S9	LA English
S10	PY 2000–2022
S11	S1 OR S2
S12	S3 OR S4
S13	S5 OR S6
S14	S11 AND S12 AND S13
S15	S14 NOT S7
S16	S8 OR S9
S17	S15 AND S16
S18	S10 AND S17

### 1.3

**Table:**

PsycINFO Search (performed on February 21st, 2022)	
S1	MAINSUBJECT.EXACT.EXPLODE ("Quality of Care") OR MAINSUBJECT.EXACT ("Quality Control“)
S2	Ti,AB (“quality of care” OR ("quality indicator" OR "quality indicators") OR ("quality measure" OR "quality measurement" OR "quality measurements" OR "quality measures") OR "quality marker*" OR ("quality improvement" OR "quality improvements") OR “quality appraisal*” OR certification* OR ("performance indicator" OR "performance indicators") OR ("performance metric" OR "performance metrics") ("performance measure" OR "performance measured" OR "performance measurement" OR "performance measurements" OR "performance measures") OR “standard* of care”)
S3	MAINSUBJECT.EXACT.EXPLODE ("Pediatrics") OR MAINSUBJECT.EXACT.EXPLODE ("Adolescent Health“)
S4	Ti,AB (child* OR infan* OR adolescen* OR ("new born" OR "new borns") OR baby OR babies OR neonat* OR perinat* OR postnat* OR ("school child" OR "school childcare" OR "school children") OR toddler* OR teen* OR boy* OR girl* OR youth* OR kindergar* OR pediatric* OR paediatric* OR school* OR preschool* OR ("pre school" OR "pre schooler" OR "pre schoolers" OR "pre schools") OR ("elementary school" OR "elementary schoolchildren" OR "elementary schoolhome" OR "elementary schooling" OR "elementary schoolk" OR "elementary schools" OR "elementary schoolteacher") OR highschool* OR ("high school" OR "high schooler" OR "high schoolers" OR "high schoolin" OR "high schooling" OR "high schools" OR "high schoolthe") OR schoolage* OR ("school age" OR "school aged" OR "school ages“))
S5	MAINSUBJECT.EXACT.EXPLODE ("Neoplasms“)
S6	Ti,AB (cancer* OR oncolog* OR neoplasm* OR carcinom* OR tumor* OR tumour* OR malignan* OR hematooncological OR “hemato oncological” OR “hematologic neoplasm*” OR hematolo*)
S7	LA (english)
S8	LA (german)
S9	YR (2000‐2022)
S10	S1 OR S2
S11	S3 OR S4
S12	S5 OR S6
S13	S10 AND S11 AND S12
S14	S7 OR S8
S15	S13 AND S14
S16	S15 AND S9
S17	S16 AND (stype.exact (“Scholarly Journals”)
S18	S17 AND PEER (yes))

## APPENDIX 2

### 2.1 Websites accessed within the gray literature search.

**Table:**

Website	Reason for exclusion or comment	Quality criteria extracted from the website
https://www.aonnonline.org/31‐aonn/223‐aonn‐evidence‐based‐navigation‐metrics	Did not clearly define, mention, or apply quality criteria for pediatric oncology	No
https://ukneqas.org.uk/	Addressed laboratory procedures	No
NQF: Quality Positioning System™ (qualityforum.org)	Reasons for exclusion of criteria: < 75% of the diagnosis were cancer diagnosis, < 75% of target population were aged ≤18 years, or too specific	No
https://www.stjude.org/global/sjcares/profile.html	No response to an email request (Contacted to request the PrOFILE Tool)	No
https://www.nice.org.uk/guidance/qs55/chapter/Quality‐statement‐1‐Multidisciplinary‐teams‐for‐young‐people	Retrieved one report from the website (NICE, 2014)	Yes, from a report (NICE, 2014) found on this website

## APPENDIX 3

### 3.1 Numbers and proportions of excluded criteria by exclusion reason.

**Table:**

Exclusion reason	Number of excluded criteria	Proportion of all excluded criteria (*n* = 329) in %
Too broad (e.g., not clear how to measure the fulfillment of criteria objectively)	113	34.35
Too specific (e.g., for a specific treatment center or diagnosis)	75	22.8
Does not measure the quality of care or at least not at only an individual center	59	17.93
Covered by good clinical practice	17	5.17
Covered by clinical trial participation or treatment protocols	29	8.81
Regulated nationally	23	6.99
Related to the certification of professionals or specialties	8	2.43
Given by MDT (e.g., “In case of a necessary interim consultation about a patient an extra meeting of the MDT with all its members should be arranged within one working day).” (Knops et al., 2012)	3	0.91
Not an outcome of the study (e.g., “rates of chemotherapy errors” (McCavit & Winick, 2012) mentioned in the discussion of an article but not in the results or major topic of this article)	2	0.61
Total	329	100
