# Supplementary material for: Quality criteria for pediatric oncology centers: A systematic literature review
Source: Cancer Med. 2023 Aug 16;12(18):18999–9012. doi: 10.1002/cam4.6452 (PMC10557895; doi:10.1002/cam4.6452)
Supplement: Supplementary file 1 — Data S1 [file CAM4-12-18999-s001.pdf]

Supporting Information 1 Quality assessments of included publications using the critical appraisal tools of the Joanna Briggs Institute (JBI) (https://jbi.global/critical-appraisal-tools)

**CHECKLIST FOR SYSTEMATIC REVIEWS AND RESEARCH SYNTHESES**												
First author or publisher (Year)	Is the review question clearly and explicitly stated?	Were the inclusion criteria appropriate for the review question?	Was the search strategy appropriate?	Were the sources and resources used to search for studies adequate?	Were the criteria for appraising studies appropriate?	Was critical appraisal conducted by two or more reviewers independently?	Were there methods to minimize errors in data extraction?	Were the methods used to combine studies appropriate?	Was the likelihood of publication bias assessed?	Were recommendations for policy and/or practice supported by the reported data?	Were the specific directives for new research appropriate?	**Total quality category**
IQWiG (2005, 2009)	yes	yes	yes	yes	yes	unclear	yes	yes	not applicable	yes	yes	Quality 2
Bradley (2013)	yes	yes	yes	yes	unclear	unclear	unclear	not applicable	not applicable	yes	yes	Quality 3
Knops (2012)	yes	yes	yes	yes	unclear	unclear	no	yes	not applicable	yes	yes	Quality 3
Knops (2013)	yes	yes	yes	yes	yes	yes	yes	yes	not applicable	yes	yes	Quality 1
**CHECKLIST FOR QUALITATIVE RESEARCH**												
First author or publisher (Year)	Is there congruity between the stated philosophical perspective and the research methodology?	Is there congruity between the research methodology and the research question or objectives?	Is there congruity between the research methodology and the methods used to collect data?	Is there congruity between the research methodology and the representation and analysis of data?	Is there congruity between the research methodology and the interpretation of results?	Is there a statement locating the researcher culturally or theoretically?	Is the influence of the researcher on the research, and vice-versa, addressed?	Are participants, and their voices, adequately represented?	Is the research ethical according to current criteria or, for recent studies, and is there evidence of ethical approval by an appropriate body?	Do the conclusions drawn in the research report flow from the analysis, or interpretation, of the data?	**Total quality category**	
Bradley (2013)	yes	yes	yes	yes	yes	not applicable	not applicable	yes	yes	yes	Quality 1	
Knops (2012)	yes	yes	yes	yes	yes	not applicable	no	yes	yes	yes	Quality 2	
Teichman (2017)	yes	yes	unclear	yes	yes	not applicable	not applicable	yes	yes	yes	Quality 2	
ten Berg (2018)	yes	yes	unclear	unclear	yes	not applicable	yes	unclear	yes	yes	Quality 3	
**CHECKLIST FOR COHORT STUDIES**												
First author or publisher (Year)	Were the two groups similar and recruited from the	Were the exposures measured similarly to assign	Was the exposure measured in a valid and	Were confounding factors identified?	Were strategies to deal with confounding	Were the groups/participants free of the outcome at the start of the	Were the outcomes measured in a valid and	Was the follow up time reported and sufficient to be long	Was follow up complete, and if not, were the	Were strategies to address incomplete	Was appropriate statistical analysis used?	**Total quality category**

	same population?	people to both exposed and unexposed groups?	reliable way?		factors stated?	study (or at the moment of exposure)?	reliable way?	enough for outcomes to occur?	reasons to loss to follow up described and explored?	follow up utilized?		
de Rojas (2019)	not applicable	not applicable	unclear	unclear	unclear	not applicable	yes	yes	yes	yes	yes	Quality 3
Fletcher (2013)	not applicable	not applicable	unclear	yes	yes	not applicable	yes	not applicable	yes	not applicable	yes	Quality 2
ten Berg (2018)	not applicable	not applicable	yes	no	yes	not applicable	unclear	not applicable	no	not applicable	yes	Quality 3
**CHECKLIST FOR ANALYTICAL CROSS SECTIONAL STUDIES**												
First author or publisher (Year)	Were the criteria for inclusion in the sample clearly defined?	Were the study subjects and the setting described in detail?	Was the exposure measured in a valid and reliable way?	Were objective, standard criteria used for measurement of the condition?	Were confounding factors identified?	Were strategies to deal with confounding factors stated?	Were the outcomes measured in a valid and reliable way?	Was appropriate statistical analysis used?	**Total quality category**			
McCavit & Winick (2012)	yes	yes	not applicable	yes	no	no	yes	yes	Quality 2			
