# Supplementary material for: Quality criteria for pediatric oncology centers: A systematic literature review
Source: Cancer Med. 2023 Aug 16;12(18):18999–9012. doi: 10.1002/cam4.6452 (PMC10557895; doi:10.1002/cam4.6452)
Supplement: Supplementary file 2 — Data S2: [file CAM4-12-18999-s002.pdf]

Supporting Information 2 Included quality criteria in their original detailed version

**Publisher or first author (year)** †	**Original criterion**	**Description or measurement**	**Summarized criterion/criteria**
**Facilities and networks**			
G-BA (2021)[1]	Pharmacy with centralized cytostatic preparation available on a daily basis if needed.	yes/no	Pharmacy
Hord (2014)[2]	A pharmacy capable of accurate, well-monitored preparation and dispensing of antineoplastic agents and investigational agents		Pharmacy
Knops (2012)[3]	In a paediatric oncology centre, the following facilities are daily, including weekends, available between 8.00 and 18.00 o'clock:<br>Pharmacy with central preparation of cytostatics<br>Microbiology laboratory<br>Pathology laboratory (for morphologic, immunohistochemic and genetic tumour diagnostics)<br>Hospital catering		Pharmacy<br>Laboratories
G-BA (2021)[1]	Hematology laboratory with the possibility of cytological blood and bone marrow examinations with special cytochemical stains	yes/no	Laboratories
Hord (2014)[2]	An established relationship with a hematopathology laboratory capable of performing cell-phenotype analysis using flow cytometry, immunohistochemistry, molecular diagnosis, cytogenetics, and polymerase chain reaction–based methodology and fluorescence in situ hybridization		Laboratories
Hord (2014)[2]	A clinical chemistry laboratory with the capability to monitor antibiotic and antineoplastic drug concentration		Laboratories
Hord (2014)[2]	A blood bank capable of providing a full range of products, including irradiated and leuko-depleted blood components		Laboratories
Kowalczyk (2009)[4]	Requirements of a Paediatric Haematology and/or Oncology Unit	The unit should have similar availability of blood products especially blood, platelets, and commonly-used protein fractions.	Laboratories
G-BA (2021)[1]	Laboratory Medicine or Clinical Chemistry Laboratory	yes/no	Laboratories
G-BA (2021)[1]	Transfusion Medicine	yes/no	Laboratories
G-BA (2021)[1]	Microbiology Institute	yes/no	Microbiology Institute
G-BA (2021)[1]	Cardiology	yes/no	Pediatric cardiology
G-BA (2021)[1]	Internal hematology and oncology	yes/no	Adult hematology and oncology

G-BA (2021)[1]	The facilities required for emergency care (intensive care facility, emergency laboratory, transfusion medicine, conventional X-ray diagnostics and sonography; CT or MRI) are provided at the center	yes/no	Pediatric intensive care unit<br>Laboratories<br>Pediatric radiology
G-BA (2021)[1]	Intensive care facility for pediatric patients accessible without patient transport outside the hospital's own grounds (with capability for mechanical ventilation and acute renal replacement procedures; and blood exchange or leukapheresis)	yes/no	Pediatric intensive care unit
Hord (2014)[2]	Up-to-date diagnostic imaging facilities to perform radiography, computed tomography, MRI, ultra-sonography, radionuclide imaging, and angiography; positron-emission tomography scanning and other emerging technologies are desirable		Pediatric radiology
G-BA (2021)[1]	Advanced imaging diagnostics with the option of anesthesia/sedation examinations (accessible without patient transport outside the clinic's own premises)	yes/no	Pediatric radiology
G-BA (2021)[1]	Institute of Pathology	yes/no	Pathology
G-BA (2021)[1]	Hospital Hygiene	yes/no	Hospital hygiene
G-BA (2021)[1]	Orthopedics	yes/no	Orthopedics
G-BA (2021)[1]	Nuclear Medicine Clinic	yes/no	Nuclear medicine
DKG (2022/2021)[5]	Reporting cases to childhood cancer registry/central database	Indicator target: reporting of all primary cases to the national childhood cancer registry.<br>Numerator: primary cases of the denominator reported to the childhood cancer registry<br>Denominator: primary cases with national residency.<br>Target: ≥ 95%	Childhood cancer registry
Kowalczyk (2009)[4]	National Register of Childhood Cancer	Population-based cancer registration	Childhood cancer registry
Knops (2012)[3]	In a paediatric oncology centre the diagnoses that belong specifically to the field of paediatric oncology will be reported to a central database.	Recommendation	Childhood cancer registry
Hord (2014)[2]	Access to stem cell transplant services with the capability or availability of HLA antigen typing		Stem cell transplant unit
Hord (2014)[2]	Access within the community to pediatric hemodialysis and/or hemofiltration and apheres		Pediatric nephrology
G-BA (2021)[1]	Nephrology with dialysis	yes/no	Pediatric nephrology
Hord (2014)[2]	Access to up-to-date radiationtherapy equipment with facilities for treating pediatric patients		Radiation therapy
G-BA (2021)[1]	Radiotherapy with radiooncological procedures in line with technical progress	yes/no	Radiation therapy

Olshefski (2020)[6]	Pediatric anesthesiology for radiation oncology	When radiation therapy is delivered at an adult-orientedsite outside the primary hospital: optimal anesthesia-related care for sedation of these children requires a provider with pediatric training. The cancer care index tracks patients requiring sedation for radiation therapy managed by an anesthesiologist without extensive pediatric training	Radiation therapy
G-BA (2021)[1]	Pediatric Surgery	yes/no	Pediatric surgery
G-BA (2021)[1]	Surgery	yes/no	Pediatric surgery
Kowalczyk (2009)[4]	Requirements of a Paediatric Haematology and/or Oncology Unit	Immediate access at all times to paediatric surgery, neurosurgery and other specialties	Pediatric surgery<br>Pediatric neurosurgery
G-BA (2021)[1]	Neurosurgery	yes/no	Pediatric neurosurgery
Knops (2012)[3]	In a paediatric oncology centre, the following facilities are 24 h/day available:<br>Paediatric intensive care unit, reachable within 10 min<br>Extensive possibilities for imaging, like conventional X-rays, ultra sound, CT-scan/magnetic resonance imaging with the possibility for anaesthetics<br>Anaesthetics for diagnostic procedures or therapeutic interventions<br>Clinical chemistry laboratory<br>Specialized haematology laboratory for morphological analysis, genotyping and immunophenotyping<br>Transfusion medicine and laboratory<br>Kidney dialysis		Pediatric intensive care unit<br>Pediatric radiology<br>Pediatric anaesthetics<br>Laboratories<br>Pediatric nephrology
**Multidisciplinary team (MDT) and other experts**			
NICE (2005)[7]	Evidence that MDTs are established in each principal treatment centre and shared care centres		MDT established, including regularly scheduled MDT conferences
NICE (2005)[7]	Evidence that MDTs are established in each principal treatment centre		MDT established, including regularly scheduled MDT conferences
G-BA (2021)[1]	There is close and structured cooperation within the multiprofessional team, the results of which are documented.	fulfilled/not fulfilled	MDT established, including regularly scheduled MDT conferences
DKG (2022/2021)[5]	Presentation of multiprofessional team	Indicator target: As complete as possible presentation of the center cases in the multiprofessional team.<br><br>Numerator: center cases of the denominator that	MDT established, including regularly scheduled MDT conferences

		were presented in the multiprofessional team<br>Denominator: center cases<br><br>Target: ≥ 95%	
Knops (2012)[3]	A paediatric oncology centre has so called ‘Treatment Multidisciplinary Teams’ (TMDT) at its disposal for the treatment of children with cancer directed at the following tumour types: central nervous system (CNS) tumours, solid tumours (outside the CNS), retinoblastoma, lymphomas and leukaemia’s (including allogenic bone marrow transplantation). The TMDTs are in charge of the care for the patient and consist of representatives of all relevant care providers.	Recommendation	MDT established, including regularly scheduled MDT conferences
Kowalczyk (2009)[4]	The rights of the hospitalised child	A multi-disciplinary treatment team	MDT established, including regularly scheduled MDT conferences
NICE (2005)[7]	Protocols for referral to specialist MDTs		MDT established, including regularly scheduled MDT conferences
G-BA (2021)[1]	Each patient is presented in an internal departmental meeting in the multiprofessional team and the treatment is strategically determined	yes/no	MDT established, including regularly scheduled MDT conferences
Hord (2014)[2]	A regularly scheduled multidisciplinary care conference		MDT established, including regularly scheduled MDT conferences
Bradley (2013b)[8]	Interdisciplinary Team Meetings	Indicator Rationale: Identifies the existence of regularly scheduled interdisciplinary team meetings in which nursing, medical and behavioural professionals assemble to collectively review the progress of patients and develop a comprehensive care plan addressing all needs of patients and families. Such care plans should be documented. An interdisciplinary team meeting is distinct from a tumour board.<br>Definition: The proportion of pediatric oncology tertiary hospitals that have regularly scheduled interdisciplinary pediatric oncology team meetings to discuss patient care issues resulting in a written record.<br>Indicator Specification: Proportion<br>Numerator: Number of pediatric oncology tertiary	MDT established, including regularly scheduled MDT conferences

		centres that have regularly scheduled interdisciplinary pediatric oncology team meetings that produce a written record.<br>Denominator: Number of pediatric oncology tertiary hospitals.	
NICE (2005)[7]	Staff attendance at the MDT meetings		MDT established, including regularly scheduled MDT conferences
NICE (2005)[7]	Adequate provision of specialist staff for every MDT		Number of pediatric oncology disciplines with multidisciplinary staffing ratios for pediatric oncology
Bradley (2013b)[8]	Sufficient Multidisciplinary stuff	Indicator Rationale: Provides a measure of access to comprehensive, multidisciplinary pediatric oncology teams. Pediatric Oncology Group of Ontario (POGO)'s staffing ratio recommendations are based on the critical importance of a comprehensive, multidisciplinary team-based approach to optimal outcomes in pediatric oncology practice.<br><br>Definition:<br>a) Compliance with/ achievement of POGO recommended pediatric oncology discipline specific staffing ratios of patients to discipline.<br>b) The proportion of professional pediatric oncology disciplines (for which POGO recommended multidisciplinary staffing ratios for pediatric oncology exist) for which the POGO-recommended staffing ratios have been met.<br>Indicator Specifications:<br>a) Ratios of pediatric oncology patients to the number of discipline-specific full-time equivalent (FTE) positions for each of the 10 disciplines with POGO-recommended ratios.<br>b) Proportion<br>Numerator:<br>Number of professional pediatric oncology disciplines for which POGO-recommended multidisciplinary staffing ratios for pediatric	Number of pediatric oncology disciplines with multidisciplinary staffing ratios for pediatric oncology

		oncology have been met.<br>Denominator: Number of professional pediatric oncology disciplines for which POGO-recommended multidisciplinary staffing ratios for pediatric oncology exist (i.e. 10 disciplines)	
Bradley (2013a)[9]	Sufficient multidisciplinary staff	Indicator Rationale: Provides a measure of access to comprehensive, multidisciplinary pediatric oncology teams. POGO's staffing ratio recommendations are based on the critical importance of a comprehensive, multidisciplinary team-based approach to optimal outcomes in pediatric oncology practice.<br><br>Definition:<br>a) Compliance with/ achievement of POGOrecommended pediatric oncology discipline specific staffing ratios of patients to discipline.<br>b) The proportion of professional pediatric oncology disciplines (for which POGOrecommended multidisciplinary staffing ratios for pediatric oncology exist) for which the POGO-recommended staffing ratios have been met.<br>Indicator Specifications:<br>a) Ratios of pediatric oncology patients to the number of discipline-specific full-time equivalent (FTE) positions for each of the 10 disciplines with POGO-recommended ratios.<br>b) Proportion<br>Numerator:<br>Number of professional pediatric oncology disciplines for which POGO-recommended multidisciplinary staffing ratios for pediatric oncology have been met.<br>Denominator: Number of professional pediatric oncology disciplines for which POGO-recommended multidisciplinary staffing ratios for pediatric oncology exist (i.e. 10 disciplines)	Number of pediatric oncology disciplines with multidisciplinary staffing ratios for pediatric oncology
G-BA (2021)[1]	The multiprofessional team consists of at least the medical service, nursing service and psychosocial service and, if	fulfilled/not fulfilled	Number of pediatric oncology disciplines with multidisciplinary staffing ratios for pediatric

	necessary, the dietary/nutritional service and physiotherapy/occupational therapy		oncology<br>Pediatric oncologists<br>Pediatric oncology nurses<br>Psychosocial care/services<br>Dieticians<br>Occupational therapists
Kowalczyk (2009)[4]	Recommended staffing levels for the paediatric haematology/oncology ward	Staffing levels: based on the annual average activity, including bed occupancy, day ward attendees and clinics, should include:<br>1. a head paediatric oncologist and head nurse with appropriate deputies;<br>2. doctors appropriate to the figures outlined below;<br>3. adequate nurses to cover the workload including a link nurse who provides the link<br>between the treating unit, parents and the local community;<br>4. psychology service;<br>5. social workers – numbers determined by patient workload;<br>6. ward teachers;<br>7. activity/play therapy;<br>8. physiotherapy and occupational therapy staff;<br>9. appropriate laboratory technicians;<br>10. medical secretaries and data managers;<br>11. rehabilitation specialists;<br>12. dieticians.	Number of pediatric oncology disciplines with multidisciplinary staffing ratios for pediatric oncology<br>Pediatric oncologists<br>Pediatric oncology nurses<br>Psychosocial care/services<br>Ward teachers<br>Activity/play therapy staff<br>Occupational therapists<br>Laboratory technicians<br>Medical secretaries and data managers<br>Rehabilitation specialists<br>Dieticians
Knops (2012)[3]	A paediatric oncology centre has a functioning supportive Multidisciplinary Team at its disposal directed at the psychosocial care for paediatric oncology patients and their families. This psychosocial MDT consists at least of child psychologists, play therapists, social workers, paediatric oncology nurses, educational service workers, paediatric oncologists and if desired other specialists of the Treatment Multidisciplinary Team.	Recommendation	Number of pediatric oncology disciplines with multidisciplinary staffing ratios for pediatric oncology<br>Psychosocial care/services<br>Activity/play therapy staff<br>Pediatric oncology nurses<br>Ward teachers<br>Pediatric oncologists
Knops (2012)[3]	A paediatric oncology centre has a functioning supportive Multidisciplinary Team at its disposal directed at palliative care. This palliative care MDT consists at least of a case manager, general practitioner, district nurse, specialized paediatrician and nurse for palliative care, child psychologist and paediatric	Recommendation	Number of pediatric oncology disciplines with multidisciplinary staffing ratios for pediatric oncology<br>Pediatric oncology nurses

	oncologist. The palliative care MDT is involved with every patient for whom cure is not feasible.		Palliative care specialists<br>Pediatric oncologists<br>Psychosocial care/services
Knops (2012)[3]	A paediatric oncology centre has a functioning supportive Multidisciplinary Team at its disposal directed at the management of pain in children. This pain MDT consists at least of a paediatric oncologist, paediatric nurse, play therapist, paediatric neurologist and a paediatric anaesthesiologist for complex pain management.	Recommendation	Number of pediatric oncology disciplines with multidisciplinary staffing ratios for pediatric oncology<br>Pain management experts<br>Pediatric oncologists<br>Pediatric oncology nurses<br>Activity/play therapy staff<br>Pediatric neurologist<br>Pediatric anesthesiology
Knops (2012)[3]	A treatment MDT has a logistical chain for executing and processing diagnostic biopsies. In this logistical chain the following members participate: paediatric neurosurgeon, intervention radiologist, paediatric anaesthesiologist, paediatric intensivist, paediatric pathologists and experts in the field of tumor morphology, genetics and immunology.	Recommendation	Number of pediatric oncology disciplines with multidisciplinary staffing ratios for pediatric oncology Pediatric anesthesiology<br>Pediatric radiologists<br>Pediatric anesthesiology<br>Genetics specialists<br>Pediatric critical care specialists<br>Pediatric pathologist<br><br>Facilities and Networks: Pathology<br>Facilities and Networks: Pediatric intensive care unit
Knops (2012)[3]	A paediatric oncology centre has a functioning supportive Multidisciplinary Team at its disposal directed at the follow-up care for childhood cancer survivors. This follow-up MDT consists at least of a paediatric oncologist, medical oncologist (adult care), radiotherapist, paediatric surgeon, paediatric neurosurgeon, paediatric neurologist, case manager, nurse practitioner, psychologist, education expert and employment expert.	Recommendation	Number of pediatric oncology disciplines with multidisciplinary staffing ratios for pediatric oncology<br>Pediatric oncologists<br>Pediatric surgeons<br>Pediatric neurologist<br>Pediatric oncology nurses<br>Psychosocial care/services<br>Ward teachers<br>Occupational therapists

			Facilities and Networks:<br>Neurosurgery<br>Facilities and Networks:<br>Radiation therapy
NICE (2005)[7]	Suggested core attendance of multidisciplinary team (MDT) members at principal treatment centres during the care pathway.<br>**Diagnosis**<br>Oncologist/haematologist<br>Radiologist<br>Surgeon/neurosurgeon<br>Pathologist/cytogeneticist<br>Clinical oncologist<br><br>**Treatment**<br>Treating oncologist<br>Key worker<br>Paediatric haematologist<br>Specialist nurses<br>Nurses from inpatient and day care units<br>Specialist pharmacist<br>Dietitian and other appropriate allied health professionals<br>Paediatric oncology or other speciality outreach nurse/key worker<br><br>**Psychosocial support**<br>Treating oncologist and haematologist<br>Key worker<br>Play specialist; activity coordinator/youth worker<br>Psychological services professional<br>Specialist outreach nurse<br>Appropriate allied health professionals<br>Teacher<br>Social worker<br>Nurses from inpatient and day care units<br><br>**Palliative care**<br>Lead clinician<br>Key worker		Number of pediatric oncology disciplines with multidisciplinary staffing ratios for pediatric oncology<br>Pediatric oncologists<br>Pediatric radiologists<br>Pediatric surgeon<br>Pediatric oncology nurses<br>Pharmacist experienced in chemotherapy preparation<br>Psychosocial care/services<br>Ward teachers<br>Activity/play therapy staff<br>Dieticians<br>Palliative care specialist<br>Pediatric pathologist<br><br>Facilities and Networks:<br>Pediatric neurosurgery

	Palliative care specialist/oncologist/haematologist<br>Social worker<br>Specialist outreach nurse<br>Specialist pharmacist<br>Psychological services professional<br>Appropriate allied health professional		
Knops (2012)[3]	In a paediatric oncology centre, the following caregivers are present on working days between 8.00 and 18.00 o'clock, outside these hours, they are available for consultation and can be present within 60 min:<br>Paediatric oncologist<br>Paediatric surgeon<br>Paediatric neurosurgeon<br>Paediatric intensivist<br>Paediatric radiotherapist<br>Paediatric neurologist<br>Paediatric anaesthesiologist<br>Paediatric cardiologist<br>Paediatric nephrologist<br>Paediatric gastroenterologist<br>Paediatric radiologist<br>Paediatric pulmonologist<br>Ear–nose–throat specialist<br>Ophthalmologist	Recommendation	Pediatric oncologist<br>Pediatric surgeons<br>Pediatric critical care specialists<br>Radiation oncologists<br>Pediatric neurologist<br>Pediatric anesthesiology<br>Pediatric cardiologist<br>Pediatric nephrologist<br>Pediatric gastroenterologist<br>Pediatric radiologists<br>Pediatric pulmonologist<br>Ear–nose–throat specialist<br>Ophthalmologist<br><br>Facilities and networks:<br>Pediatric neurosurgery<br>Facilities and networks:<br>Intensive care unit<br>Facilities and networks:<br>Radiation therapy
Knops (2012)[3]	The paediatric oncology MDT designates a practitioner-in-charge and its substitute who act on behalf of the paediatric oncology MDT. The practitioner-in-charge and its substitute are documented in the (digital) medical file and are known to the patient, parents and/or guardians.		Pediatric oncology practitioner-in-charge/lead clinician (also with expertise in late effects)
NICE (2005)[7]	Suggested core attendance of multidisciplinary team (MDT) members responsible for the care of patients following completion of treatment.<br>**Late effects MDT**<br>Lead clinician (oncologist with expertise in late effects)<br>Key worker<br>Specialist nurse<br>Endocrinologist		Pediatric oncology practitioner-in-charge/lead clinician (also with expertise in late effects)<br>Pediatric oncology nurses<br>Pediatric endocrinologist<br>Psychosocial care/services

	Appropriate allied health professional<br>Psychological services professional		
Hord (2014)[2]	Board-certified pediatric hematologists/oncologists		Pediatric oncologists
Hord (2014)[2]	Pediatric oncology nurses who are certified in chemotherapy, knowledgeable about pediatric protocols, and experienced in the management of complications of therapy; Association of Pediatric Hematology/ Oncology Nurses certification is preferable		Pediatric oncology nurses
Hord (2014)[2]	Board-certified radiologists with specific expertise as evidenced by certification and training in the diagnostic imaging and radiologic intervention of infants, children, adolescents, and young adults		Pediatric radiologists
Hord (2014)[2]	Board-certified surgeons with expertise in pediatric general surgery		Pediatric surgeons
Hord (2014)[2]	Dentists who have completed additional training in pediatric dentistry		Dentist
Hord (2014)[2]	A board-certified radiation oncologist trained and experienced in the treatment of infants, children, and adolescent		Radiation oncologists
Hord (2014)[2]	Board-certified pediatric medical subspecialists available to participate actively in all areas of the care of the child with cancer, including anesthesiology, critical care, infectious diseases, cardiology, neurology, endocrinology and metabolism, genetics, gastroenterology, child and adolescent psychiatry, nephrology, palliative medicine, pulmonology, [...], and behavioral and developmental specialists		Pediatric anesthesiology<br>Pediatric critical care specialists<br>Pediatric infectious diseases specialists<br>Pediatric cardiologist<br>Pediatric neurologist<br>Pediatric endocrinologist<br>Genetics specialists<br>Pediatric gastroenterologist<br>Psychosocial care/services<br>Pediatric nephrologist<br>Palliative care specialists<br>Pediatric pulmonologist
Hord (2014)[2]	Pediatric physical and mental rehabilitation services, including pediatric physiatry and pediatric psychiatry		Psychosocial care/services<br>Rehabilitation specialists
Hord (2014)[2]	Experts with knowledge in complementary and alternative therapies		Complementary and alternative therapies
Hord (2014)[2]	Nutrition experts with experience in pediatrics and the capability of preparing, administering, and monitoring total parenteral nutrition		Dieticians
Hord (2014)[2]	Pharmacist(s) with experience and training in providing chemotherapy and supportive care medicines for pediatric		Pharmacists experienced in chemotherapy preparation

	patients with cancer; a board-certified clinical pharmacologist, where available, may be helpful in patient management		
Hord (2014)[2]	Access to instruction and learning activities for hospitalized children and adolescents		Ward teachers
Hord (2014)[2]	Pathologists with special expertise as evidenced by certification and training in the pathology of hematologic malignancies, tumors of the central nervous system, and solid tumors in children, adolescents, and young adults		Pediatric pathologist
**Supportive care**			
**Supportive care: Central venous catheter (CVC)**			
Olshefski (2020)[6]	Surgical complication related to Central Venous Catheter Placement (CVC) placement	Effective communication to the surgical team regarding the intended device type is essential to ensure that the correct device is implanted. Furthermore, it is key to ensure that the central venous catheter (CVC) tip is in an acceptable location for long-term use. Failure to insert the desired device or leaving the catheter tip in an unacceptable location within the heart or great vessels counts as a Cancer Care Index event	Surgical complication rates (failure to insert the desired device or leaving the catheter tip in an unacceptable location) related to CVC placement
Bradley (2013b)[8]	Central Venous Line (CVL) Infection Rate	Indicator Rationale: Indexes a common but potentially serious complication of use of CVLs for fluid, drug and blood product administration. CVLs are commonly used in pediatric cancer patients and infection in this patient population is particularly hazardous.<br>Definition: The number of confirmed central venous line (CVL)-derived primary blood stream infection cases amongst pediatric oncology patients, per 1,000 pediatric oncology central venous line days.<br>Indicator Specification: Rate<br>Numerator: Number of confirmed CVL-derived primary blood stream infection cases amongst pediatric oncology patients with central venous lines over 12-month period, multiplied by 1,000.<br>Denominator: Number of pediatric oncology CVL days over the same 12-month period	Complication rates: particularly the incidence of CVC-associated infection
NICE (2005)[7]	Complication rates, particularly incidence of (central venous catheter) CVC-associated infection		Complication rates: particularly the incidence of CVC-associated infection
**Supportive care: CVC and supportive care (guidelines) and febrile neutropenia (F&N)**			

Bradley (2013b)[8]	Supportive care guidelines	Existence of supportive care guidelines<br>Written policies/ procedures for the management of CVC	Existence of supportive care guidelines<br>Written policies/ procedures for the management of CVC<br>Guidelines on how to approach a child with F&N (availability, risk-stratified approach, escalation for fever persistence)
Bradley (2013a)[9]	Supportive care guidelines	Indicator Rationale: Provides a baseline measure of whether formal policies are in place for management of four key supportive care issues in pediatric oncology. As primary treatment modalities have intensified, so too has the need for formalized supportive care guidelines.<br>Inconsistent and variable supportive care can add substantially to the burden of illness.<br><br>Definition: The proportion of pediatric oncology tertiary and/or Satellite programs that have formal, written policies/ procedures for the management of:<br>a) fever/ neutropenia (in both tertiary and Satellite centres);<br>b) ambulatory management of fever/ neutropenia (in the tertiary centres only);<br>c) central venous line management (in both tertiary and Satellite centres);<br>d) anti-neoplastic induced nausea and vomiting (in both tertiary and Satellite centres)<br>Indicator Specification: Proportion<br>Numerator: Number of tertiary and/ or Satellite pediatric oncology programs who have formal supportive care guidelines for the management of each of the above four supportive care issues for children with cancer.<br>Denominator: Number of tertiary and/ or Satellite pediatric oncology programs.	Existence of supportive care guidelines<br>Written policies/ procedures for the management of CVC<br>Guidelines on how to approach a child with F&N (availability, risk-stratified approach, escalation for fever persistence)
**Supportive care: Supportive care (guidelines)**			
Knops (2012)[3]	A paediatric oncology centre has written guidelines about diagnostics and treatment of all paediatric cancer types,	Recommendation	Existence of supportive care guidelines<br>Supportive care (guidelines for):

	supportive care (including pain management), follow-up care after the end of treatment and palliative care.		Pain relief, including local protocol for pain relief procedures and adequate pain management<br>Supportive care (guidelines) for: Palliative care (including bereavement)
NICE (2005)[7]	Written protocol in management of nausea, vomiting and bowel disturbance in use with evidence of multidisciplinary input and regular review		Supportive care (guidelines for): nausea, vomiting and bowel disturbance
NICE (2005)[7]	Compliance with and effectiveness of protocol (Management of nausea, vomiting and bowel disturbance)		Supportive care (guidelines for): nausea, vomiting and bowel disturbance
Olshefski (2020)[6]	Nutritional assessment	Detailed survey of the patient`s nutritional status by a nutritional expert [….] should be performed before initiating a therapy, which provides a baseline to manage utrition throughout treatment. Not documenting this assessment constitutes an event	Supportive care (guidelines for): Nutritional assessment
Bradley (2013b)[8]	Guidelines for Nutritional Support	Indicator Rationale: Proposed as a high-level indicator of best practice regarding nutritional support for children with cancer. Appropriate nutritional support influences outcome and quality of life for pediatric cancer patients.<br>Definition: The proportion of pediatric oncology tertiary centres that utilize a standardized guideline for nutritional support for pediatric oncology patients.<br>Indicator Specification: Proportion<br>Numerator: Number of pediatric oncology tertiary programs in Ontario that utilize a standardized guideline for nutritional support of pediatric cancer patients.<br>Denominator: Number of pediatric oncology tertiary centres in Ontario.	Supportive care (guidelines for): Nutritional assessment
NICE (2005)[7]	Provision of protocols detailing measures to ensure adequate nutritional support		Supportive care (guidelines for): Nutritional assessment
NICE (2005)[7]	Nutritional status of patients		Supportive care (guidelines for): Nutritional assessment
Knops (2012)[3]	The caregiver makes assistance in nutrition, also for the home-situation, available.	Recommendation	Supportive care (guidelines for): Nutritional assessment

NICE (2005)[7]	Evidence that there are effective dental screening and treatment protocols available before and during treatment		Supportive care (guidelines for): Dental care
NICE (2005)[7]	Palliative Care		Supportive care (guidelines for): Palliative care (including bereavement)
Kowalczyk (2009)[4]	Palliative Care		Supportive care (guidelines for): Palliative care (including bereavement)
Olshefski (2020)[6]	Palliative care consult	…in patients with progressive cancer, the goal is to provide palliative care options at least 30 days before death; if palliative care or hospice referral , including a face-to-face consultation, was not initiated within that time frame, it becomes a Cancer Care Index event; as with influenza vaccine administration, if a family refuses a consultation with a palliative care physician, it is counted as an event because..., it is the responsibility of the oncologist to make the case for palliative care when appropriate	Supportive care (guidelines for): Palliative care (including bereavement)
de Rojas (2019)[10]	% of relapsed patients receiving support from the local Palliative Care Unit		Supportive care (guidelines for): Palliative care (including bereavement)
Bradley (2013b)[8]	Patients Referred for End-of-Life Interlink Care	Indicator Rationale: Provides an index of the coordination of hospital and community-based resources to effect optimal end-of-life care.<br><br>Definition: The proportion of pediatric oncology patients, 0 to 17 years of age, who are referred to the Interlink nursing service for end-of-life care.<br>Indicator Specification: Proportion<br>Numerator: Number of pediatric oncology patients, 0 to 17 years of age, who are referred to Interlink at the time of end-of-life care.<br>Denominator: Number of pediatric oncology patients, 0 to 17 years of age, who die.	Supportive care (guidelines for): Palliative care (including bereavement)
Knops (2012)[3]	The physician-in-charge and the case manager of palliative care make a palliative care scheme based on values, goals, needs and developmental stage of the patient and its family, in accordance with law.	Recommendation	Supportive care (guidelines for): Palliative care (including bereavement)

Knops (2012)[3]	The caregiver emphasizes that optimal palliative care will be given when cure is no longer feasible.	Recommendation	Supportive care (guidelines for): Palliative care (including bereavement)
Knops (2012)[3]	The caregiver provides for the family of deceased children a program for dealing with bereavement.	Recommendation	Supportive care (guidelines for): Palliative care (including bereavement)
NICE (2005)[7]	Bereavement		Supportive care (guidelines for): Palliative care (including bereavement)
NICE (2005)[7]	Rehabilitation		Supportive care (guidelines for): (Neuro-) Rehabilitation
Kowalczyk (2009)[4]	Rehabilitation		Supportive care (guidelines for): (Neuro-) Rehabilitation
NICE (2005)[7]	Documented referral policies to guide referral for rehabilitation		Supportive care (guidelines for): (Neuro-) Rehabilitation
NICE (2014)[11]	Structure:<br>Evidence of local arrangements to ensure that all children [...] who have had a central nervous system malignancy receive a specialist neuro-rehabilitation care package.		Supportive care (guidelines for): (Neuro-) Rehabilitation
NICE (2014)[11]	Process:<br>The proportion of children [...] who have had treatment for a central nervous system malignancy who receive a specialist neuro-rehabilitation care package.	Numerator – the number of people in the denominator receiving a specialist neuro-rehabilitation care package.<br>Denominator – the number of children [...] who have had treatment for a central nervous system malignancy.	Supportive care (guidelines for): (Neuro-) Rehabilitation
NICE (2014)[11]	Process:<br>The proportion of children [...] with cancer who are assessed for potential future fertility problems and advised about their options for fertility preservation before treatment is started.	Numerator – the number of people in the denominator who are assessed for potential future fertility problems before treatment and are advised about their options for fertility preservation.<br>Denominator – the number of children [...] diagnosed with cancer.	Supportive care (guidelines for): Fertility (preservation) discussion
NICE (2014)[11]	Structure:<br>Evidence of local arrangements to ensure that children [...] with cancer are assessed for potential future fertility problems and advised about their options for fertility preservation before treatment is started.		Supportive care (guidelines for): Fertility (preservation) discussion
Knops (2012)[3]	The caregiver actively offers the possibility of fertility preservation to boys as well as girls.	Recommendation	Supportive care (guidelines for): Fertility (preservation) discussion

Olshefski (2020)[6]	Fertility discussion	Discussing implications of recommended treatment regarding fertility is required in the consent discussion and must be documented in the electronic medical record, or it counts as a Cancer Care Index event	Supportive care (guidelines for): Fertility (preservation) discussion
NICE (2005)[7]	Written protocols for pain relief procedures		Supportive care (guidelines for): Pain relief, including local protocol for pain relief procedures and adequate pain management
NICE (2005)[7]	Evidence that specialist and age-appropriate pain relief services are available when required		Supportive care (guidelines for): Pain relief, including local protocol for pain relief procedures and adequate pain management
Teichman (2017)[12]	Pain management	Population: all patients Outcome: mean/median score on survey question, “how frequently did your health care provider ask you about the pain your child was experiencing?” Respondents choose from 4 options (never/rarely/at most visits/at every visit). Measurement: 6-mo audit of all surveys. Responses converted to a 1-4 scale	Supportive care (guidelines for): Pain relief, including local protocol for pain relief procedures and adequate pain management
IQWiG (2005)[13]	Pain	(can be benchmarked)	Supportive care (guidelines for): Pain relief, including local protocol for pain relief procedures and adequate pain management
Bradley (2013b)[8]	Access to Expert Management of Pain Control	Indicator Rationale: Measures access to expert pain management, or pain teams, for the control of acute or chronic pain in children with cancer at the tertiary pediatric oncology program level. Pain in children with cancer can be a frequent occurrence and difficult to control and therefore may require specialized expertise through access to comprehensive pain management care services. Definition: The proportion of pediatric oncology programs that have access to expert pain management, defined as a formal, consistent resource group or expert staff member for consultation and/or management of acute or	Supportive care (guidelines for): Pain relief, including local protocol for pain relief procedures and adequate pain management

		chronic pain for children with cancer.<br>Indicator Specification: Proportion<br>Numerator: Number of hospital survey respondents in the tertiary pediatric oncology programs who have access to a formal, consistent expert resource group or staff member for consultation and/ or management of acute or chronic pain for children with cancer.<br>Denominator: Number of survey respondents in the tertiary pediatric oncology programs	
Bradley (2013a)[9]	Access to expert pain management	Indicator Rationale: Measures access to expert pain management, or pain teams, for the control of acute or chronic pain in children with cancer at the tertiary pediatric oncology program level. Pain in children with cancer can be a frequent occurrence and difficult to control and therefore may require specialized expertise through access to comprehensive pain management care services.<br>Definition: The proportion of pediatric oncology programs that have access to expert pain management, defined as a formal, consistent resource group or expert staff member for consultation and/or management of acute or chronic pain for children with cancer.<br>Indicator Specification: Proportion<br>Numerator: Number of hospital survey respondents in the tertiary pediatric oncology programs who have access to a formal, consistent expert resource group or staff member for consultation and/ or management of acute or chronic pain for children with cancer.<br>Denominator: Number of survey respondents in the tertiary pediatric oncology programs	Supportive care (guidelines for): Pain relief, including local protocol for pain relief procedures and adequate pain management
IQWiG (2005)[13]	Procedures for psychosocial support and rehabilitation		Supportive care (guidelines for): Psychological or psychosocial care, including provision of/information about social care
NICE (2005)[7]	Psychosocial care		Supportive care (guidelines for): Psychological or psychosocial

			care, including provision of/information about social care
NICE (2014)[11]	Structure:<br>b) Evidence of local arrangements to ensure that children [...] with cancer, and their families and carers, can access services delivering psychological and social support		Supportive care (guidelines for): Psychological or psychosocial care, including provision of/information about social care
G-BA (2021)[1]	It (psychosocial service) consists of employees of the:<br>- psychological-psychotherapeutic area	fulfilled/not fulfilled	Supportive care (guidelines for): Psychological or psychosocial care, including provision of/information about social care
DKG (2022/2021)[5]	Counseling by the Psychosocial Service (PSD)	Indicator target: As complete as possible counseling of patients and families by the psychosocial service.<br>Numerator: Center cases of the denominator or their families who have been counselled by the psychosocial service.<br>Denominator: center cases<br>Target: ≥ 95%	Supportive care (guidelines for): Psychological or psychosocial care, including provision of/information about social care
Kowalczyk (2009)[4]	Psychological and psychosocial care		Supportive care (guidelines for): Psychological or psychosocial care, including provision of/information about social care
Olshefski (2020)[6]	Psychology referral	Each patient should be referred to psychology and social work and an education specialist, when age appropriate, who performs a needs assessment;<br>If referral in any of these areas does not occur, it becomes a Cancer Care Index event	Supportive care (guidelines for): Psychological or psychosocial care, including provision of/information about social care
Knops (2012)[3]	The caregiver provides specialized psychosocial and (neuro-)psychological care depending on the nature of the problems.	Recommendation	Supportive care (guidelines for): Psychological or psychosocial care, including provision of/information about social care
G-BA (2021)[1]	It (psychosocial service) consists of employees of the:<br>- and the socio-pedagogical-social work area	fulfilled/not fulfilled	Supportive care (guidelines for): Psychological or psychosocial care, including provision of/information about social care
Kowalczyk (2009)[4]	Social care		Supportive care (guidelines for): Psychological or psychosocial care, including provision of/information about social care

Olshefski (2020)[6]	Social work referral	Each patient should be referred to psychology and social work and an education specialist, when age appropriate, who performs a needs assessment;<br>If referral in any of these areas does not occur, it becomes a Cancer Care Index event	Supportive care (guidelines for): Psychological or psychosocial care, including provision of/information about social care
Kowalczyk (2009)[4]	The rights of the hospitalised child	Play and education facilities	Supportive care (guidelines for): Provision of school education
Kowalczyk (2009)[4]	Education		Supportive care (guidelines for): Provision of school education
Olshefski (2020)[6]	Education needs assessments	Each patient should be referred to psychology and social work and an education specialist, when age appropriate, who performs a needs assessment;<br>If referral in any of these areas does not occur, it becomes a Cancer Care Index event	Supportive care (guidelines for): Provision of school education
Knops (2012)[3]	A paediatric oncology centre provides educational services during all stages of care	Recommendation	Supportive care (guidelines for): Provision of cancer education
Hord (2014)[2]	A formal program for cancer education for the patient, family, and or caregiver and instruction on self-management		Supportive care (guidelines for): Provision of cancer education
**Supportive care: Febrile neutropenia (F&N)**			
NICE (2005)[7]	Development of national guidelines on febrile neutropenia		Guidelines on how to approach a child with F&N (availability, risk-stratified approach, escalation for fever persistence)
NICE (2005)[7]	Development of risk-stratified protocols for the management of febrile neutropenia		Guidelines on how to approach a child with F&N (availability, risk-stratified approach, escalation for fever persistence)
NICE (2005)[7]	Development of protocols for outpatient treatment of febrile neutropenia		Guidelines on how to approach a child with F&N (availability, risk-stratified approach, escalation for fever persistence)
NICE (2005)[7]	Compliance with protocols and guidelines (febrile neutropenia)		Guidelines on how to approach a child with F&N (availability, risk-stratified approach, escalation for fever persistence)
ten Berg (2018)[14]	Having a general recommendation on the antimicrobial policy of febrile neutropenia in children with cancer<br>1. No recommendation<br>2. Verbal agreement<br>3. Written recommendation in own document system		Guidelines on how to approach a child with F&N (availability, risk-stratified approach, escalation for fever persistence)

	4. According to the Dutch Childhood Oncology Group (DCOG) guideline		
ten Berg (2018)[14]	Percentage of febrile neutropenia episodes without microbial focus, which are treated with ceftazidim	Numerator: The number of febrile neutropenia episodes without microbial focus, for which patients received ceftazidim according to the Dutch Childhood Oncology Group (DCOG) guideline<br>Denominator: All episodes of febrile neutropenia without microbial focus	Number/Proportion of clinical F&N episodes in which the patients with or without microbial focus are treated with first line antibiotics according to local guidelines
ten Berg (2018)[14]	The percentage of clinical febrile neutropenia episodes in children with cancer, in which a patient is admitted to the intensive care unit (ICU)	Numerator: The number of clinical febrile neutropenia episodes in children with cancer, in which a patient is admitted to the ICU<br>Denominator: All clinical febrile neutropenia episodes	Number/Proportion of clinical F&N episodes in which patients are admitted to ICU
ten Berg (2018)[14]	The percentage of clinical febrile neutropenia episodes of which patients have died	Numerator: The number of clinical febrile neutropenia episodes of which patients have died<br>Denominator: The total number of clinical febrile neutropenia episodes	Number/Proportion of clinical F&N episodes in which the patient died
Olshefski (2020)[6]	Fungal Health Care-Associated Infections (HAI)	…infections are tracked and included in the Cancer Care Index if hospital acquired	Fungal Health Care-Associated Infections (HAI)
Fletcher (2013)[15]	Time to antibiotics (TTA) of 60 minutes	…we propose 60 minutes as the TTA benchmark for pediatric patients with febrile neutropenia<br>TTA: defined as the time in minutes from presentation to either triage (emergency department [ED]), registration (outpatient clinic), or admitting (direct admission) to the first dose of parenteral antibiotics.<br>Measured in 60-minute intervals and as comtinous variable	Time to antibiotic (TTA) administration
McCavit & Winick (2012)[16]	Time-to-Antibiotic Administration (TTA)	Abstract: Nearly half of respondents track TTA. Most reported using a benchmark of less than 60 min from arrival. TTA is a commonly used quality-of-care measure for pediatric febrile neutropenia despite an absence of studies establishing its validity and a lack of data supporting its impact on outcomes of febrile neutropenia.<br><br>...as the time required for the administration of the first dose of empiric antibiotics in children with febrile neutropenia. It was implied that the TTA measurement began upon the patient's arrival at	Time to antibiotic (TTA) administration

		the institution, but this was not explicitly stated.<br><br>...as a Quality of Care Measure in Children With Febrile Neutropenia<br>most clinics using it as a quality measure set <60 min as a threshold<br><br>Results: Forty-five percent [...] of respondents reported tracking<br>TTA as a quality-of-care measure [...]. Of those, over 90% reported using a TTA standard/benchmark of <30 or <60 min and use the same TTA standard/benchmark for the outpatient clinic, the ED, and the inpatient unit.<br><br>But: ...further investigation of its validity as a measure of the process and outcomes of care are warranted before it is accepted as a standard quality-of-care measure in the pediatric oncology community	
Olshefski (2020)[6]	Time to Antibiotics for fever/neutropenia	best practice requires prompt antibiotic administration to febrile patients with neutropenia - within 60 minutes after emergency department or hospital arrival, rather than from the time when the absolute neutrophil count is determined<br>if that time exceeds 60 minutes, it counts as a Cancer Care Index event	Time to antibiotic (TTA) administration
Corey & Snyder (2008)[17]	Time to antibiotics 30-minute door/fever-to-patient delivery (30-minute door/fever-to-patient antibiotics delivery)	…a goal of 30 minutes was chosen for the initiation of STAT antibiotics to a febrile neutropenic patient admission or new-onset fever and was to be achieved at a rate of 95%	Time to antibiotic (TTA) administration
**Treatment**			
NICE (2005)[7]	Rates of refusal and failure to complete treatment – annual report		Number/Proportion of refusal and failure to complete treatment
NICE (2005)[7]	Compliance with chemotherapy protocols		Protocol compliance (e.g., number of major clinical trial protocol violations)

NICE (2005)[7]	Protocol compliance by both patients and clinical specialists		Protocol compliance (e.g., number of major clinical trial protocol violations)
Bradley (2013b)[8]	Major Clinical Trial Protocol Violation	Indicator Rationale: Provides an externally assessed<br>standardized measure of compliance with clinical trial specification and good clinical practice.<br>Definition: The proportion of patients on Children's Oncology Group (COG) or Dana Farber Cancer Institute (DFCI) clinical trials who have a documented major clinical trial protocol violation, such as an error in timing of a step in treatment or investigation<br>Indicator Specification: Proportion<br>Numerator: Number of COG or DFCI major clinical trial protocol violations.<br>Denominator: Number of pediatric cancer patients enrolled on COG or DFCI clinical trials.	Protocol compliance (e.g., number of major clinical trial protocol violations)
de Rojas (2019)[10]	Chemotherapy (CT) plan deviations		Protocol compliance (e.g., number of major clinical trial protocol violations)
NICE (2014)[11]	Structure:<br>Evidence of local arrangements to ensure that children [...] are assessed for eligibility for relevant clinical trials and offered the opportunity to take part.		Number/Proportion of clinical trial participation
NICE (2014)[11]	Process:<br>a) The proportion of children [...] with cancer and eligible for a clinical trial who are offered the opportunity to take part.	Numerator – the number of people in the denominator offered the opportunity to take part.<br>Denominator – children [...] with cancer and eligible for a clinical<br>trial.	Number/Proportion of clinical trial participation
Knops (2012)[3]	The physician-in-charge offers to children, who are eligible, participation in clinical trials. If children do not participate in a clinical trial and if there is no (inter)national guideline available, an individual treatment scheme will be developed based on maximal scientific proof and expertise.	Recommendation	Number/Proportion of clinical trial participation
NICE (2014)[11]	Process:<br>b) The proportion of children [...] with cancer who are recruited	Numerator – the number of people in the denominator recruited into the clinical trial.<br>Denominator – the number of children [...] with cancer and eligible for a clinical trial.	Number/Proportion of clinical trial participation

DKG (2022/2021)[5]	Included center cases in therapy optimization studies/German Society for Pediatric Oncology and Hematology (GPOH) registries.	Indicator target: As complete as possible inclusion of center cases in therapy optimization studies/GPOH registries.<br>Numerator: center cases included in therapy optimization studies/GPOH registries.<br>Denominator: primary cases with national residence.<br>Target: ≥ 90%	Number/Proportion of clinical trial participation
NICE (2005)[7]	Surveys of numbers of children and young people who are entered into available clinical trials		Number/Proportion of clinical trial participation
Bradley (2013a)[9]	Clinical trial participation	Indicator Rationale: This indicator indexes the extent to which Ontario's treatment centres are aligned with what is universally accepted as the gold standard of care, recognizing that there may be many reasons for non-participation in a clinical trial. Comparison to similar indicators in American and European groups will provide a benchmark.<br><br>Definition: The proportion of newly diagnosed pediatric cancer cases, 0 to 17 years of age inclusive, diagnosed and treated in a pediatric cancer centre in Ontario, who are enrolled on a Research Ethics Board (REB) approved, cancer therapeutic clinical trial.<br>Excludes enrolments on biology, registry, or observational studies.<br>Indicator Specification: Proportion.<br>Numerator: Total number of newly diagnosed pediatric oncology cases, 0 to 17 years of age inclusive, diagnosed and treated at a pediatric cancer centre in Ontario, who are enrolled on a REB-approved, cancer therapeutic clinical trial.<br>Denominator: Total number of newly diagnosed pediatric oncology cases, 0 to 17 years of age, diagnosed and treated at a pediatric cancer centre in Ontario.	Number/Proportion of clinical trial participation
de Rojas (2019)[10]	% of patients enrolled in clinical trials as salvage treatment		Number/Proportion of clinical trial participation
Bradley (2013b)[8]	Clinical Trial Participation	Indicator Rationale: This indicator indexes the extent to which Ontario's treatment centres are	Number/Proportion of clinical trial participation

		aligned with what is universally accepted as the gold standard of care, recognizing that there may be many reasons for non-participation in a clinical trial. Comparison to similar indicators in American and European groups will provide a benchmark.<br><br>Definition: The proportion of newly diagnosed pediatric cancer cases, 0 to 17 years of age inclusive, diagnosed and treated in a pediatric cancer centre in Ontario, who are enrolled on a Research Ethics Board (REB) approved, cancer therapeutic clinical trial.<br>Excludes enrolments on biology, registry, or observational studies.<br>Indicator Specification: Proportion.<br>Numerator: Total number of newly diagnosed pediatric oncology cases, 0 to 17 years of age inclusive, diagnosed and treated at a pediatric cancer centre in Ontario, who are enrolled on a REB-approved, cancer therapeutic clinical trial.<br>Denominator: Total number of newly diagnosed pediatric oncology cases, 0 to 17 years of age, diagnosed and treated at a pediatric cancer centre in Ontario.	
de Rojas (2019)[10]	% of patients enrolled in clinical trials as frontline treatment		Number/Proportion of clinical trial participation
DKG (2022/2021)[5]	Therapy deviation from tumor conference recommendation	Target: Deviation from the recommendation of the tumor conference as seldom as possible.<br>Numerator: Center cases of the denominator in which there was at least one deviation from the therapy recommendation(s) of the tumor conference.<br>Denominator: center cases that were presented at the interdisciplinary tumor conference<br>Target ≤ 5%	Number/Proportion of patients presented in the interdisciplinary tumor conference (for solid and liquid tumors separately or combined)
G-BA (2021)[1]	If the patient needs to be cared for by several disciplines, he or she will also be presented in an interdisciplinary tumor conference	yes/no	Number/Proportion of patients presented in the interdisciplinary tumor conference (for solid and liquid tumors separately or combined)

G-BA (2021)[1]	The result of the interdisciplinary tumor conference is documented	yes/no	Number/Proportion of patients presented in the interdisciplinary tumor conference (for solid and liquid tumors separately or combined)
Hord (2014)[2]	A regularly scheduled multidisciplinary pediatric tumor board		Number/Proportion of patients presented in the interdisciplinary tumor conference (for solid and liquid tumors separately or combined)
Bradley (2013b)[8]	Tumour Boards	Indicator Rationale: This indicator will measure the availability of regularly scheduled tumour boards. A tumour board is a multidisciplinary treatmentplanning forum in which a number of physicians who are experts in different specialties review and discuss the medical condition and treatment options of a patient. A tumour board review may include the expert input of a pediatric oncologist, a surgeon, a pathologist, a diagnostic imaging specialist and a radiation oncologist. The main purpose of the tumour board is to ensure that all appropriate diagnostic tests,<br>treatment options, and the most appropriate treatment recommendations are generated for each cancer patient. The discussions of the tumor board should be recorded.<br>Definition: The proportion of pediatric oncology tertiary hospitals that have regularly scheduled multidisciplinary pediatric oncology tumour boards that produce a written record.<br>Indicator Specification: Proportion<br>Numerator: Number of pediatric oncology tertiary hospitals that have regularly scheduled multidisciplinary pediatric oncology tumour boards that produce a written record.<br>Denominator: Number of pediatric oncology tertiary hospitals.	Number/Proportion of patients presented in the interdisciplinary tumor conference (for solid and liquid tumors separately or combined)
DKG (2022/2021)[5]	Presentation of interdisciplinary tumor conference	Indicator target: As complete as possible presentation of center cases (main group II-XII) in the interdisciplinary tumor conference.	Number/Proportion of patients presented in the interdisciplinary tumor conference (for solid and

		Numerator: center cases of the denominator that were presented in the interdisciplinary tumor conference<br>Denominator: center cases main group II-XII (without main group I).<br>Target ≥ 95%	liquid tumors separately or combined)
**Treatment: Delay in/ Wait time to start of**			
NICE (2005)[7]	Delays to start of radiotherapy		Delay in/ Wait time to start of Radiotherapy
Bradley (2013b)[8]	Chemotherapy Admission Delay	Indicator Rationale: This indicator will index the frequency with which chemotherapy administration has to be deferred for resource reasons, measured over a specified and reasonable time frame. No absolute benchmark exists, but the presumption that a delay of greater than 72 hours is not acceptable accords with clinical trial protocol specifications.<br><br>Definition: The proportion of admissions for chemotherapy that are delayed for greater than 72 hours from the time the patient is deemed ready for admission, due to a lack of hospital bed (i.e. hospital resource) availability.<br>Indicator Specification: Proportion<br>Numerator: Number of pediatric oncology admissions for chemotherapy that are delayed for greater than 72 hours from the time the patient is deemed ready for admission.<br>Denominator: Number of pediatric oncology admissions for chemotherapy.	Delay in/ Wait time to start of: Chemotherapy
Bradley (2013a)[9]	Chemotherapy admission delay	Indicator Rationale: This indicator will index the frequency with which chemotherapy administration has to be deferred for resource reasons, measured over a specified and reasonable time frame. No absolute benchmark exists, but the presumption that a delay of greater than 72 hours is not acceptable accords with clinical trial protocol specifications.<br><br>Definition: The proportion of admissions for	Delay in/ Wait time to start of: Chemotherapy

		chemotherapy that are delayed for greater than 72 hours from the time the patient is deemed ready for admission, due to a lack of hospital bed (i.e. hospital resource) availability.<br>Indicator Specification: Proportion<br>Numerator: Number of pediatric oncology admissions for chemotherapy that are delayed for greater than 72 hours from the time the patient is deemed ready for admission.<br>Denominator: Number of pediatric oncology admissions for chemotherapy.	
NICE (2005)[7]	Delays to start of planned chemotherapy		Delay in/ Wait time to start of: Chemotherapy
Bradley (2013b)[8]	First Therapeutic Intervention Wait Time	Indicator Rationale: Provides a measure of efficacy of implementation of treatment following a definitive diagnosis and will allow comparison between Provincial values and individual tertiary centres. If differences exist, they may provide evidence and support requests for incremental resource support.<br>Definition: The number of days between the date of diagnosis and the date of first therapeutic intervention. The date of diagnosis is the date on which the definitive procedure confirming the diagnosis was carried out.<br>The date of first therapeutic intervention is the date on which the first therapeutic intervention (i.e. chemotherapy, radiotherapy, or surgery) was initiated.<br>Indicator Specification: Median, Range, and 90th percentile	Delay in/ Wait time to start of: First therapeutic intervention
Bradley (2013a)[9]	First therapeutic intervention wait time	Indicator Rationale: Provides a measure of efficacy of implementation of treatment following a definitive diagnosis and will allow comparison between Provincial values and individual tertiary centres. If differences exist, they may provide evidence and support requests for incremental resource support.<br>Definition: The number of days between the date of diagnosis and the date of first therapeutic intervention. The date of diagnosis is the date on which the definitive procedure confirming the	Delay in/ Wait time to start of: First therapeutic intervention

		diagnosis was carried out.<br>The date of first therapeutic intervention is the date on which the first therapeutic intervention (i.e. chemotherapy, radiotherapy, or surgery) was initiated.<br>Indicator Specification: Median, Range, and 90th percentile	
NICE (2005)[7]	Time taken for the production of pathology reports		Delay in/ Wait time to start of release of pathology results
Bradley (2013b)[8]	Time Taken for the Production of Pathology Reports	Indicator Rationale: This indicator will measure the turnaround time within the diagnostic labs to enable the start of appropriate therapy. It will allow comparison to a benchmark<br>Definition: The number of days between the date that a definitive diagnostic specimen is received by the pathology department to the date a definitive pathology report is issued.<br>Indicator Specification: Mean, Median, Interquartile Range	Delay in/ Wait time to start of release of pathology results
Bradley (2013a)[9]	Time to pathology report production	Indicator Rationale: This indicator will measure the turnaround time within the diagnostic labs to enable the start of appropriate therapy. It will allow comparison to a benchmark<br>Definition: The number of days between the date that a definitive diagnostic specimen is received by the pathology department to the date a definitive pathology report is issued.<br>Indicator Specification: Mean, Median, Interquartile Range	Delay in/ Wait time to start of release of pathology results
**Treatment: Medication**			
NICE (2014)[11]	Outcome:<br>The number of patient safety incidents in children [...] related to chemotherapy prescriptions.		Number/Proportion of patient safety incidents related to chemotherapy prescriptions
Bradley (2013b)[8]	Actual Drug/ Dose Errors	Indicator Rationale: Pediatric oncology patients on active therapy are prescribed a range of medications including drugs with significant toxicity and narrow therapeutic margins. This indicator will provide an index of the frequency with which actual medication errors occur (i.e. the incorrect drug or dose is administered to the patient).	Number/Proportion of actual drug or dose errors identified for patients on active treatment

		Definition: The number of actual drug or dose errors identified for pediatric oncology patients on active treatment.<br>Indicator Specification: Proportion<br>Numerator: Number of actual drug or dose errors that were identified for pediatric oncology patients on active treatment.<br>Denominator: Number of pediatric oncology patients on active treatment.	
Bradley (2013a)[9]	Actual drug/dose errors	Indicator Rationale: Pediatric oncology patients on active therapy are prescribed a range of medications including drugs with significant toxicity and narrow therapeutic margins. This indicator will provide an index of the frequency with which actual medication errors occur (i.e. the incorrect drug or dose is administered to the patient).<br>Definition: The number of actual drug or dose errors identified for pediatric oncology patients on active treatment.<br>Indicator Specification: Proportion<br>Numerator: Number of actual drug or dose errors that were identified for pediatric oncology patients on active treatment.<br>Denominator: Number of pediatric oncology patients on active treatment.	Number/Proportion of actual drug or dose errors identified for patients on active treatment
NICE (2005)[7]	Errors and near misses		Number/Proportion of actual drug or dose errors identified for patients on active treatment
NICE (2005)[7]	Incident reporting of errors and near misses		Number/Proportion of actual drug or dose errors identified for patients on active treatment
Bradley (2013a)[9]	Potential drug/dose errors	Indicator Rationale: Pediatric oncology patients on active therapy are prescribed a range of medications including drugs with significant toxicity and narrow therapeutic margins. This indicator will provide an index of the frequency with which potential medication errors occur (i.e. the error is detected before being administered to the patient).<br>Definition: The number of potential drug or dose errors (i.e. near misses) that were identified prior to	Number/Proportion of potential drug or dose errors identified for patients on active treatment

		administration in pediatric oncology patients on active treatment.<br>Indicator Specification: Proportion<br>Numerator: Number of potential drug or dose errors that were identified prior to administration for pediatric oncology patients on active treatment.<br>Denominator: Number of pediatric oncology patients on active treatment.	
Bradley (2013b)[8]	Potential Drug/ Dose Errors (Near Misses)	Indicator Rationale: Pediatric oncology patients on active therapy are prescribed a range of medications including drugs with significant toxicity and narrow therapeutic margins. This indicator will provide an index of the frequency with which potential medication errors occur (i.e. the error is detected before being administered to the patient).<br>Definition: The number of potential drug or dose errors (i.e. near misses) that were identified prior to administration in pediatric oncology patients on active treatment.<br>Indicator Specification: Proportion<br>Numerator: Number of potential drug or dose errors that were identified prior to administration for pediatric oncology patients on active treatment.<br>Denominator: Number of pediatric oncology patients on active treatment.	Number/Proportion of potential drug or dose errors identified for patients on active treatment
NICE (2005)[7]	Prescribing errors		Number/Proportion of potential drug or dose errors identified for patients on active treatment
Bradley (2013a)[9]	Wait time: Sedation for ambulatory procedures	Indicator Rationale: The lack of timely access to anesthesia services can delay diagnostic procedures, surgical interventions, radiation delivery, and routine intrathecal chemotherapy. This indicator will index the frequency with which non-emergent, ambulatory pediatric oncology procedures<br>requiring anesthesia are deferred until the next day or beyond due to<br>lack of availability of anesthesia services. Deferral of ambulatory<br>procedures until the next day or beyond places an	Number/Proportion of elective pediatric oncology ambulatory procedures requiring anesthesia that are deferred to the next day or beyond due to resource limitation(s)

		additional burden<br>on patients and families as another visit to the hospital is required.<br><br>Definition: The proportion of elective pediatric oncology<br>ambulatory procedures requiring anesthesia deferred<br>to the next day or beyond due to hospital resource limitation(s).<br>Indicator Specification: Proportion<br>Numerator: Number of elective pediatric oncology ambulatory procedures requiring anesthesia that are deferred to the next day or beyond due to resource limitation(s).<br>Denominator: Number of elective pediatric oncology ambulatory procedures requiring anesthesia.	
Bradley (2013b)[8]	Wait Time for Ambulatory Procedures Requiring Sedation	Indicator Rationale: The lack of timely access to anesthesia services can delay diagnostic procedures, surgical interventions, radiation delivery, and routine intrathecal chemotherapy. This indicator will index the frequency with which non-emergent, ambulatory pediatric oncology procedures<br>requiring anesthesia are deferred until the next day or beyond due to<br>lack of availability of anesthesia services. Deferral of ambulatory procedures until the next day or beyond places an additional burden<br>on patients and families as another visit to the hospital is required.<br><br>Definition: The proportion of elective pediatric oncology ambulatory procedures requiring anesthesia deferred to the next day or beyond due to hospital resource limitation(s).<br>Indicator Specification: Proportion<br>Numerator: Number of elective pediatric oncology ambulatory procedures requiring anesthesia that are deferred to the next day or beyond due to	Number/Proportion of elective pediatric oncology ambulatory procedures requiring anesthesia that are deferred to the next day or beyond due to resource limitation(s)

		resource limitation(s).<br>Denominator: Number of elective pediatric oncology ambulatory procedures requiring anesthesia.	
**Long-term care**			
Kowalczyk (2009)[4]	Requirements of a Paediatric Haematology and/or Oncology Unit	There should be systems in place once treatment has finished in order to monitor<br>long-term outcomes.	Established follow-up structure
Kowalczyk (2009)[4]	Monitoring the late outcomes of cancer		Established follow-up structure
NICE(2005)[7]	Appropriate follow-up of patients at risk of late effects		Established follow-up structure
NICE (2005)[7]	Documented local protocols for the continued care and follow up of patients including identification of key workers for individual patients		Established follow-up structure
Bradley (2013b)[8]	Eligible Survivors Enrolled in AfterCare	Indicator Rationale: Provides a measure of access to and utilization of the provincial pediatric oncology AfterCare program, which provides systematic follow-up of survivors of childhood cancer across all ages and continuing surveillance for early detection of/ intervention in late effects of treatment, as well as health promotion and education.<br><br>Definition:<br>a) The proportion of pediatric survivors of pediatric cancer, 0 to 17 years of age, eligible for AfterCare who are enrolled in the program.<br>b) The proportion of adult survivors of pediatric cancer, 18 years of age and older, eligible for AfterCare who are enrolled in the program.<br>Survivors are considered eligible for AfterCare two years after the completion of all therapy, provided they are disease-free and have not relapsed.<br>Survivors are considered enrolled in AfterCare after referral plus attendance at first visit.<br>Indicator Specification: Proportion<br>Numerator:<br>a) Number of pediatric survivors of childhood cancer, 0 to 17 years of age, enrolled in AfterCare.<br>b) Number of adult survivors of childhood cancer, 18 years of age and older, enrolled in AfterCare.<br>Denominator:	Established follow-up structure

		a) Number of pediatric survivors of childhood cancer, 0 to 17 years of age, eligible for enrollment in AfterCare.<br>b) Number of adult survivors of childhood cancer, 18 years of age and older, eligible for enrollment in AfterCare.	
Bradley (2013a)[9]	Eligible survivors enrolled in AfterCare	Indicator Rationale: Provides a measure of access to and utilization of the provincial pediatric oncology AfterCare program, which provides systematic follow-up of survivors of childhood cancer across all ages and continuing surveillance for early detection of/ intervention in late effects of treatment, as well as health promotion and education.<br><br>Definition:<br>a) The proportion of pediatric survivors of pediatric cancer, 0 to 17 years of age, eligible for AfterCare who are enrolled in the program.<br>b) The proportion of adult survivors of pediatric cancer, 18 years of age and older, eligible for AfterCare who are enrolled in the program.<br>Survivors are considered eligible for AfterCare two years after the completion of all therapy, provided they are disease-free and have not relapsed.<br>Survivors are considered enrolled in AfterCare after referral plus attendance at first visit.<br>Indicator Specification: Proportion<br>Numerator:<br>a) Number of pediatric survivors of childhood cancer, 0 to 17 years of age, enrolled in AfterCare.<br>b) Number of adult survivors of childhood ancer, 18 years of age and older, enrolled in AfterCare.<br>Denominator:<br>a) Number of pediatric survivors of childhood cancer, 0 to 17 years of age, eligible for enrollment in AfterCare.<br>b) Number of adult survivors of childhood cancer, 18 years of age and older, eligible for enrollment in AfterCare.	Established follow-up structure

Bradley (2013a)[9]	Survivors with a survivor care plan	Indicator Rationale: Provides a measure of the efficiency and integration of the pediatric cancer system across the trajectory of disease and age. Survivor care plans allow for the provision of knowledge to and the empowerment of survivors, facilitating self-advocacy, while enabling healthcare providers to best meet the individual needs of survivors.<br><br>Definition: The proportion of pediatric and adult survivors of childhood cancer enrolled in AfterCare with an<br>individualized and standardized survivor care plan.<br>Indicator Specification: Proportion<br>Numerator: Number of pediatric and adult survivors of childhood cancer enrolled in AfterCare who have been provided with a survivor care plan.<br>Denominator: Number of pediatric and adult survivors of childhood cancer enrolled in AfterCare.	Number/Proportion of survivors of childhood cancer with a survivor care plan
Bradley (2013b)[8]	Survivors with a Survivor Care Plan	Indicator Rationale: Provides a measure of the efficiency and integration of the pediatric cancer system across the trajectory of disease and age. Survivor care plans allow for the provision of knowledge to and the empowerment of survivors, facilitating self-advocacy, while enabling healthcare providers to best meet the individual needs of survivors.<br><br>Definition: The proportion of pediatric and adult survivors of childhood cancer enrolled in AfterCare with an<br>individualized and standardized survivor care plan.<br>Indicator Specification: Proportion<br>Numerator: Number of pediatric and adult survivors of childhood cancer enrolled in AfterCare who have been provided with a survivor care plan.<br>Denominator: Number of pediatric and adult survivors of childhood cancer enrolled in AfterCare.	Number/Proportion of survivors of childhood cancer with a survivor care plan

NICE (2014)[11]	Structure:<br>Evidence of local arrangements to ensure that children [...] who have been treated for cancer have an end-of-treatment summary and care plan that includes agreed follow-up and monitoring arrangements.		Number/Proportion of survivors of childhood cancer with a survivor care plan
NICE (2014)[11]	Process:<br>a) The proportion of children [...] completing treatment for cancer who have an end-of-treatment summary and care plan.	Numerator – the number of people in the denominator who have an end-of-treatment summary and care plan.<br>Denominator – the number of children [...] completing treatment for cancer.	Number/Proportion of survivors of childhood cancer with a survivor care plan
NICE (2014)[11]	Process:<br>b) The proportion of children [...] treated for cancer who have their end-of-treatment summary and care plan reviewed 5 years after the end of their initial treatment.	Numerator – the number of people in the denominator who have their end-of-treatment summary and care plan reviewed 5 years after the end of their initial treatment.<br>Denominator – the number of children [...] treated for cancer with an end-of-treatment summary and care plan.	Number/Proportion of survivors who have their survivorship care plan reviewed 5 years after the end of treatment
NICE (2005)[7]	Evidence of care plans for long-term care for all patients		Number/Proportion of survivors of childhood cancer with a survivor care plan
Knops (2012)[3]	The physician-in-charge and case manager of follow-up care make in collaboration with other members of the follow-up MDT and the patient a follow-up scheme at the end, one year and 5 years after treatment directed at control of the specific tumor and early detection of relapse or secondary tumours.	Recommendation	Number/Proportion of survivors of childhood cancer with a survivor care plan
Knops (2012)[3]	The physician-in-charge and case manager of follow-up care make in collaboration with other members of the follow-up MDT and the patient a follow-up scheme at the end, one year and 5 years after treatment directed at the early detection and treatment of possible side effects, psychosocial and neuropsychological problems after the treatment of childhood cancer.	Recommendation	Number/Proportion of survivors of childhood cancer with a survivor care plan
Knops (2012)[3]	The physician-in-charge makes in collaboration with the patient arrangements for transition from paediatric oncology to adult medical oncology when applicable.	Recommendation	Established transition structure
Hord (2014)[2]	An established program designed to provide long-term, multidisciplinary follow-up of successfully treated patients at the original treatment center or by a physician led team of health care professionals who are familiar with the potential adverse effects of treatment of childhood cancer; as survivors of		Established transition structure

	childhood cancer move into adulthood, transition to an adult provider may be appropriate		
**Volume and numbers**			
DKG (2022/2021)[5]	Primary cases (all first diagnoses of first and second tumors; multiple answers possible; (Pat. with first tumor + cases with second tumors)	Numerator: Primary cases<br>Denominator:--<br>Target: currently none	Number of cases per year and provider/clinic
DKG (2022/2021)[5]	Center cases (= primary cases + total cases with initial presentation with recurrence).	Numerator: Center cases<br>Denominator:--<br>Target ≥ 30	Number of cases per year and provider/clinic
Kowalczyk (2009)[4]	Requirements of a Paediatric Haematology and/or Oncology Unit	There should be a minimum number of cases that a unit sees to remain efficient and competent. This probably equates to at least 30 new cases per year.	Number of cases per year and provider/clinic
de Rojas (2019)[10]	Number of patients/year		Number of cases per year and provider/clinic
Knops (2013)[18]	Volume of a hospital	...the treatment of more than five cases per year per provider (i.e. either hospital or physician) as 'high volume'<br>...that higher volume hospitals, higher case volume providers, and specialised hospitals are related to the better outcome in paediatric oncology<br><br>Although there is no consensus about a threshold in paediatric oncology that indicates the transition from low volume providers to high volume providers, for this review we defined the treatment of more than five cases per year per provider (i.e. either hospital or physician) as 'high volume'.<br>This definition of high volume was made after reading the selected studies and based on the encountered volume numbers.	Number of cases per year and provider/clinic

† Abbreviations of publishers: G-BA Federal Joint Committee (Gemeinsamer Bundesausschuss), DKG German Cancer Society (Deutsche Krebsgesellschaft), NICE National Institute for Health and Care Excellence, IQWiG Institute for Quality and Efficiency in Health Care (Institut für Qualität und Wirtschaftlichkeit im Gesundheitswesen)

References

1. Richtlinie des Gemeinsamen Bundesausschusses über Maßnahmen zur Qualitätssicherung für die stationäre Versorgung von Kindern und Jugendlichen mit hämato-onkologischen Krankheiten gemäß § 136 Absatz 1 Satz 1 Nummer 2 SGB V für nach § 108 SGB V zugelassene Krankenhäuser. Berlin: Gemeinsamer Bundesausschuss; 2021.
2. Hord J, Feig S, Crouch G, et al. Standards for Pediatric Cancer Centers. *Pediatrics* 2014; **134**(2): 410-4.
3. Knops RR, Hulscher ME, Hermens RP, et al. High-quality care for all children with cancer. *Ann Oncol* 2012; **23**(7): 1906-11.
4. Kowalczyk J, Pritchard-Jones K, Samardakiewicz M, et al. European Standards of Care for Children with Cancer. Warsaw: European Society for Paediatric Oncology (SIOPE), 2009.
5. Erhebungsbögen DKG Krebsgesellschaft, Anlage EB Version D1.2 (Auditjahr 2022/Kennzahlenjahr 2021) Kennzahlen und Matrix Kinderonkologische Zentren. Berlin: Deutsche Krebsgesellschaft (DKG); 2021.
6. Olshefski R, Vaughan M, YoungSaleme T, et al. The Cancer Care Index: A Novel Metric to Assess Overall Performance of a Pediatric Oncology Program. *J Patient Saf* 2020; **16**(3): e120-e5.
7. Improving Outcomes in Children and Young People with Cancer. London: National Institute for Health and Clinical Excellence (NICE), 2005.
8. Bradley NM, Robinson PD, Greenberg ML, et al. Measuring the quality of a childhood cancer care delivery system: quality indicator development. *Value Health* 2013; **16**(4): 647-54.
9. Bradley NM, Robinson PD, Greenberg ML, et al. Measuring the quality of a childhood cancer care delivery system: assessing stakeholder agreement. *Value Health* 2013; **16**(4): 639-46.
10. de Rojas T, Puertas M, Bautista F, et al. Improving the quality of care in the molecular era for children and adolescents with medulloblastoma. *Clin Transl Oncol* 2019; **21**(12): 1687-98.
11. Cancer Services for Children and Young People. London: National Institute for Health Care Excellence (NICE), 2014.
12. Teichman J, Punnett A, Gupta S. Development of Quality Metrics to Evaluate Pediatric Hematologic Oncology Care in the Outpatient Setting. *J Pediatr Hematol Oncol* 2017; **39**(2): 90-6.
13. Quality of haematological and oncological care in children: Executive summary of final report V06-01, Version 1.0. Cologne: Institute for Quality and Efficiency in Health Care (IQWiG), 2005.
14. Ten Berg S, Loeffen EAH, van de Wetering MD, et al. Development of pediatric oncology supportive care indicators: Evaluation of febrile neutropenia care in the north of the Netherlands. *Pediatr Blood Cancer* 2019; **66**(2): e27504.
15. Fletcher M, Hodgkiss H, Zhang S, et al. Prompt administration of antibiotics is associated with improved outcomes in febrile neutropenia in children with cancer. *Pediatr Blood Cancer* 2013; **60**(8): 1299-306.
16. McCavit TL, Winick N. Time-to-antibiotic administration as a quality of care measure in children with febrile neutropenia: a survey of pediatric oncology centers. *Pediatr Blood Cancer* 2012; **58**(2): 303-5.
17. Corey AL, Snyder S. Antibiotics in 30 minutes or less for febrile neutropenic patients: a quality control measure in a new hospital. *J Pediatr Oncol Nurs* 2008; **25**(4): 208-12.
18. Knops RRG, van Dalen EC, Mulder RL, et al. The volume effect in paediatric oncology: a systematic review. *Ann Oncol* 2013; **24**(7): 1749-53.
